# Incidence and prevalence of Takayasu arteritis in England before and after the COVID-19 pandemic: results from RECORDER

**DOI:** 10.1093/rap/rkag078

**Published:** 2026-07-24

**Authors:** Stephanie J Lax, Mehrdad A Mizani, Megan Rutter, David Groves, Peter Lanyon, Matthew J Grainge, Sean McPhail, Peter Stilwell, Jeanette Aston, Mary Bythell, Andrew Porter, Robert T Maughan, Taryn Youngstein, Justin C Mason, Fiona A Pearce, CVD-COVID-UK/COVID-IMPACT Consortium, Alastair Proudfoot, Andrew Constantine, Dan Jones, Krishnaraj Rathod, Nida Ahmed, Richard Fitzgerald, Dan O’Connell, Rony Arafin, Sonya Babu-Narayan, Jon Shelton, Martina Slapkova, Rosie Hinchliffe, Shane Johnson, Renin Toms, Julia Townson, Ewan Birney, Moritz Gerstung, Tomas Fitzgerald, Katherine Brown, Benjamin Zuckerman, Ernest Wong, Lily Benton, Tasanee Braithwaite, Alexis Webb, Anna Stevenson, Cathie Sudlow, Fionna Chalmers, Jadene Lewis, John Nolan, Lars Murdock, Laura Sherlock, Lynn Morrice, Mehrdad Mizani, Melissa Webb, Ross Forsyth, Samaira Khan, Sandy Wibrewdc, Steffen Petersen, Thomas Bolton, Zach Welshman, Caroline Rogers, Petr Andriushchenko, Donna Wakefield, Ahmed Salih, Alistair Marsland, Alun Davies, Arunashis Sau, Costas Kallis, Francesca Cavallo, Fu Siong Ng, Hannah Whittaker, Ioanna Tzoulaki, Jennifer Quint, Juliette Unwin, Libor Pastika, Petter Brodin, Philip Stone, Safa Salim, Sarah Cook, Sarah Onida, Alistair Marsland, Andrew Thompson, Sara Holloway, Thomas Porter, Mamas Mamas, Abdel Douiri, Adejoke Oluyase, Ajay Shah, Alexandru Dregan, Amy Ronaldson, Anna Bone, Antonio Cannata, Ben Bray, Charles Wolfe, Daniel Bromage, Dominic Oliver, Elena Nikiphorou, Emeka Chukwusa, Emel Yorganci, Gareth Williams, Gayan Perera, Harry Watson, Iain Marshall, India Tunnard, Irene Higginson, Javiera Leniz Martelli, Jayati Das-Munshi, Joanna Davies, Johnny Downs, Jonathan Hudson, Katherine Sleeman, Laia Becares, Linglong Qian, Matthew O’Connell, Mevhibe Hocaoglu, Natasha Chilman, Rachel Cripps, Richard Killick, Theresa McDonagh, Vasa Curcin, Zeljko Kraljevic, Rocco Friebel, Akshay Jagadeesh, Antonio Gasparrini, Arturo de la Cruz, Dorothea Nitsch, Julian Matthewman, Neil Pearce, Patrick Bidulka, Qiuju Li, Sinéad Langan, Thiago Silva, Zhengnan Zhu, Martin Rutter, Alex Grundmann, Adam Hollings, Alistair Jones, Angeliki Antonarou, Daniel Schofield, Deborah Lowe, Elizabeth Kelly, Richardson, Humaira Hussein, Nickie Wareing, Shoaib Ali Ajaib, Mark Barber, Carole Morris, Felix Greaves, Jennifer Beveridge, Shaun Rowark, Thomas Lawrence, Vandana Ayyar-Gupta, Vahé Nafilyan, Deepti Gurdasani, Frank Kee, Paz Tayal, David Cromwell, Amar Shah, Swapna Mandal, Florian Falter, Joseph Newman, Jennifer Rossdale, Baboucarr Njie, Elijah Behr, Nuria Sanchez, Rosie Freer, Xinkai Wang, Daniel Harris, Amanda Marchant, Ashley Akbari, Daniel King, David Powell, Elizabeth A Ellins, Fatemeh Torabi, Gareth Davies, Hoda Abbasizanjani, Huw Strafford, Jane Lyons, Julian Halcox, Laura North, Marcos del Pozo Banos, Owen Pickrell, Ronan Lyons, Ann John, Robert Aldridge, Abraham Olvera-Barrios, Adnan Tufail, Alasdair Warwick, Alex Handy, Alexei Yavlinsky, Alvina Lai, Ami Banerjee, Ana Torralbo, Ana-Catarina Pinho-Gomes, Andrej Ivanovic, Andrew Lambarth, Anthony Khawaja, Ashkan Dashtban, Ashley Dickson, Becky White, Christina Pagel, Christopher Tomlinson, Chu Siyu, David Selby, Emma Whitfield, Eva Keller, Evaleen Malgapo, Ferran Espuny-Pujol, Flavien Hardy, Floriaan Schmidt, Freya Allery, Harry Hemingway, Helen Richardson, Hiroyuki Yoshimura, Honghan Wu, Jinge Wu, Johan Thygesen, Johannes Heyl, Julia Ive, Kate Cheema, Katie Harron, Ken Li, Kerrie Stevenson, Laura Pasea, Louise Choo, Luca Grieco, Manuel Gomes, Mehrdad Mizani, Mengyun Liu, Michalis Katsoulis, Mohamed Mohamed, Naomi Launders, Nushrat Khan, Paula Lorgelly, Pedro Machado, Pia Hardelid, Qi Huang, Riyaz Patel, Roy Schwartz, Rui Providencia, Ruth Gilbert, Samuel Kim, Scott Isherwood, Simon Ellershaw, Sonya Crowe, Spiros Denaxas, Stephanie Wu, Tuankasfee Hama, Waty Lilaonitkul, Wojtek Banas, Yi Mu, Yohhei Hamada, Yoryos Lyratzopoulos, David Osborn, Arun Pherwani, Mary Joan Macleod, Sarah Wang, Mark Thomas, Alejandro Gonzalez-Aquines, Arun Karthikeyan Suseeladevi, Ben Gibbison, Daniel Fudulu, Dann Mitchell, Deborah Lawler, Eleanor Walsh, Elsie Horne, Elvis Annan, Ewan Walker, Falinyi Samson, Gianni Angelini, Jeremy Chan, John Macleod, Jonathan Sterne, Joseph Okeke, Katharine Looker, Livia Pierotti, Luisa Zuccolo, Marwa Al Arab, Massimo Caputo, Mercy Nthiwa, Neil Davies, Paul Madley-Dowd, Rachel Denholm, Rochelle Knight, Rupert Payne, Shubhra Sinha, Tim Dong, Tom Palmer, Venexia Walker, Yueying Li, Yvonne Nartey, Alexia Sampri, Angela Wood, Carmen Petitjean, Chriselda Oliver, Elena Raffetti, Elias Allara, Emanuele Di Angelantonio, Eoin McKinney, Eric Harshfield, Fabian Falck, Genevieve Cezard, Hannah Harrison, Haoting Zhang, Holly Pavey, Isabel Walter, Jasmine Kiley, Jessica Barrett, John Danesh, Lisa Pennells, Lois Kim, Matilda Pitt, Mayank Dalakoti, Megan Ritson, Mike Inouye, Millie Zhou, Pearl Liu, Robert Fletcher, Rutendo Mapeta, Salma Chaudhury, Samantha Ip, Spencer Keene, Stelios Boulitsakis Logothetis, Stephen Kaptoge, Tom Pape, Wen Shi, Xilin Jiang, Xiyun Jiang, Yanfan Li, Atanu Bhattacharjee, Daniel Morales, Huan Wang, Ify Mordi, Samira Bell, Alan Carson, Alice Hosking, Athina Spiliopoulou, Baljean Dhillon, Carlos Sanchez Soriano, Caroline Jackson, Claire Tochel, Dave Yeung, Gavin Chapman, Gwenetta Curry, Helen Colhoun, Joe Mellor, Kelly Fleetwood, Laura Sherlock, Luke Blackbourn, Michelle Williams, Miguel Bernabeu Llinares, Rebecca Reynolds, Richard Chin, Steven Kerr, Tim Wilkinson, Verónica Cabreira, William Berthon, William Whiteley, John Dennis, Kerry Pearn, Michael Allen, Angela Henderson, Clea du Toit, Colin Berry, Craig Melville, Dennis Tran, Filip Sosenko, Frederick Ho, Jill Pell, Jocelyn Friday, John Cleland, Naveed Sattar, Ninian Lang, Salil Deo, Sandosh Padmanabhan, Terry Quinn, Charlotte Sturley, Jianhua Wu, Jonathan Batty, Marlous Hall, Ramesh Nadarajah, Tamara del Toro, Anna Hansell, Anvesha Singh, Cameron Razieh, Claire Lawson, Clare Gillies, Elpida Vounzoulaki, Francesco Zaccardi, Iain Squire, Kamlesh Khunti, Matthew Bown, Muhammad Rashid, Sharmin Shabnam, Shirley Sze, Tom Yates, Yogini Chudasama, João Malato, Andrew Mason, Benedict Michael, Caroline Dale, David Hughes, Francesca Zaccagnino, Giulia Ballabio, Ike Chukwudi, Maria Sudell, Mark Green, Munir Pirmohamed, Orla Hilton, Pardis Biglarbeigi, Reecha Sofat, Rohan Takhar, Ruwanthi Kolamunnage-Dona, Stephen McKeever, Bernard Keavney, Craig Smith, David Jenkins, Eva Henning, Evan Kontopantelis, George Tilston, Glen Martin, Hector Chinoy, Joseph Firth, Lana Bojanić, Matthew Sperrin, Max Lyon, Maya Buch, Ruth Norris, Sarah Steeg, Simon Williams, Steven Zhao, Zenas Yiu, Federico Fama, Marta Colaneri, Camille Carroll, Charlotte Parbery-Clark, Dexter Canoy, Precious Onyeachu, Fiona Pearce, Laila Tata, Ralph Akyea, Stephanie Lax, Aashna Uppal, Akshay Shah, Antonella Delmestri, Antony Palmer, Ben Goldacre, Ben Lacey, Dani Prieto-Alhambra, Eva Morris, Gardash Bakhishli, George Nicholson, Hayley Evans, James Sheppard, Joseph Kamtchum Tatuene, Kazem Rahimi, Linxin Li, Lucy Wright, Marta Pineda Moncusi, Mohammad Mamouei, Nick Hall, Parag Gajendragadkar, Paula Dhiman, Qingze Gu, Ramla Khan, Raph Goldacre, Richard Williams, Sara Khalid, Seb Bacon, Seyed Alireza Hasheminasab, Shishir Rao, Xiaomin Zhong, Zeinab Bidel Taleshmekaeil, Nathalie Conrad, Marie-Louise Zeissler, Norman Briffa, Suzanne Mason, Tim Chico, Nazrul Islam, Amanj Kurdi, Kim Kavanagh, Marion Bennie, Tanja Mueller, Harry Wilde, Majel McGranahan, Christina van der Feltz-Cornelis, Han-I Wang, Lorna Fraser, Tapiwa Tungamirai

**Affiliations:** Department of Lifespan and Population Health, School of Medicine, University of Nottingham, Nottingham, UK; British Heart Foundation Data Science Centre, Health Data Research UK, London, UK; Department of Lifespan and Population Health, School of Medicine, University of Nottingham, Nottingham, UK; National Disease Registration Service, NHS England, Leeds, UK; Department of Rheumatology, Nottingham University Hospitals NHS Trust, Nottingham, UK; Department of Lifespan and Population Health, School of Medicine, University of Nottingham, Nottingham, UK; National Disease Registration Service, NHS England, Leeds, UK; Department of Lifespan and Population Health, School of Medicine, University of Nottingham, Nottingham, UK; National Disease Registration Service, NHS England, Leeds, UK; Department of Rheumatology, Nottingham University Hospitals NHS Trust, Nottingham, UK; Department of Lifespan and Population Health, School of Medicine, University of Nottingham, Nottingham, UK; National Disease Registration Service, NHS England, Leeds, UK; National Disease Registration Service, NHS England, Leeds, UK; National Disease Registration Service, NHS England, Leeds, UK; National Disease Registration Service, NHS England, Leeds, UK; National Heart and Lung Institute, Imperial College London, London, UK; National Heart and Lung Institute, Imperial College London, London, UK; National Heart and Lung Institute, Imperial College London, London, UK; National Heart and Lung Institute, Imperial College London, London, UK; Department of Lifespan and Population Health, School of Medicine, University of Nottingham, Nottingham, UK; National Disease Registration Service, NHS England, Leeds, UK; Department of Rheumatology, Nottingham University Hospitals NHS Trust, Nottingham, UK

**Keywords:** Takayasu arteritis, epidemiology, incidence, prevalence, COVID-19

## Abstract

**Objectives:**

Takayasu arteritis (TAK) is a rare large vessel vasculitis associated with substantial morbidity and mortality. Evidence on its epidemiology, including the COVID-19 pandemic’s impacts, is incomplete. We aimed to describe the incidence and prevalence of TAK in England using national routinely collected healthcare data.

**Methods:**

Using Hospital Episode Statistics for the whole of England (Admitted Patient Care and Outpatients), we identified all people with the International Classification of Diseases, 10th revision code for TAK. We validated our case ascertainment and diagnosis date refinement strategies in five National Health Service Trusts and using the unique Imperial College TAK cohort of 123 confirmed cases. We calculated the incidence of TAK in England from 2006 to 2023 (cohort design), particularly focusing on the COVID-19 pandemic, and point prevalence for 1 July 2022 (cross-sectional design). Results were reported by key demographic variables.

**Results:**

The incidence of TAK in England was 1.3 (95% CI 1.3, 1.4) per million per year and was stable except for a 30% decrease in 2020–2021 (COVID-19 pandemic). The prevalence of TAK in mid-2022 was 22.5 per million (95% CI 21.3, 23.8). The median age at diagnosis was 53 years. Incidence and prevalence were both higher in women and in Asian/Asian British and Other ethnic groups. There was variation between different geographic areas, but socio-economic status did not have a marked effect.

**Conclusion:**

Nearly 1300 people have TAK in England, with >70 new cases each year apart from during the COVID-19 pandemic. These estimates, based on a 10-fold larger cohort than previous European studies, will be essential in planning and commissioning pandemic-prepared specialist services.

Key messages

- The prevalence of TAK in 2022 was 22.5 per million, with 1285 individuals currently living with it.
- The incidence of TAK in England was 1.3 per million per year, with ≈70 people newly developing TAK in England each year.
- Incidence decreased by 30% in the year 2020–2021 (COVID-19 pandemic) and was otherwise stable.

## Introduction

Takayasu arteritis (TAK) is an ultrarare large vessel vasculitis, predominantly affecting the aorta and its main branches, and is associated with significant morbidity and mortality [1, 2]. Existing evidence on the epidemiology of TAK is incomplete due to the rarity of the condition, which makes estimating the COVID-19 pandemic’s impact especially hard [3].

Previous UK epidemiology of TAK was published in 2009 using general practice (community) health records, showing an annual incidence of 0.8/million (95% CI 0.4, 1.3) and a mean prevalence of 4.7/million based on only 14 people living with the condition [4]. In 2016, tocilizumab was commissioned by National Health Service (NHS) England as a first biologic treatment for TAK in adults [5]. The commissioning policy used an annual incidence of 0.7/million (95% CI 0.3, 1.2), based on a contemporary study of the epidemiology of TAK in southern Sweden, where only 13 patients were identified between 1997 and 2011 [6]. We published a meta-analysis in 2021 that showed a global incidence of 1.11/million person-years (95% CI 0.70, 1.76), with considerable variation seen between different geographic populations, and females more commonly affected than males [7].

In this study we aimed to provide robust contemporary estimates of the incidence and prevalence of TAK in England using a validated case ascertainment strategy and population-wide electronic health records. Due to barriers to accessing national health data in England, well-described in the Sudlow review [8], we completed this study 7 years after the initial research grant was awarded. We conducted the epidemiology study as part of a COVID-focused research consortium and we particularly explored whether the pandemic influenced missed diagnoses and/or diagnostic delay. Accurate incidence and prevalence data are important when considering the health of the nation during crises such as the COVID-19 pandemic, commissioning of specialised services, funding of high-cost drugs and developing new therapeutics.

## Methods

This study was conducted in two phases. A validation phase was first conducted in partnership with the National Disease Registration Service (NDRS) at NHS England and the Imperial College vasculitis service. Then, the epidemiology study was conducted with the British Heart Foundation (BHF) Data Science Centre’s CVD-COVID-UK/COVID-IMPACT Consortium [9]. A combination of cohort and cross-sectional study design features were used to generate estimates of incidence and prevalence, respectively.

This project addresses the core research aim of the consortium to explore the indirect impact of the COVID-19 pandemic and the government and NHS response to it on the presentation, diagnosis, management and outcomes of health conditions.

### Phase 1: Validation of case ascertainment and date of diagnosis

Full details of the validation phase are presented in Supplementary Data S1 and summarised briefly here.

#### Validation of diagnostic coding

The NDRS data sharing agreements with NHS Trusts in England enabled us to confirm the diagnosis in a sample of records. Hospital Episode Statistics (HES) admitted patient care (APC) episodes between 2003 and 2017 with the International Classification of Diseases, 10th revision (ICD-10) diagnostic code for ‘Aortic arch syndrome [Takayasu]’ (M31.4) were validated in five hospital trusts (*n* = 62), with a positive predictive value of 80.6% (95% CI 68.6–89.6). The coded cases in which the diagnosis was not TAK were two unclassified vasculitis, one SAPHO (synovitis, acne, pustulosis, hyperostosis and osteitis), one former Kawasaki disease, one aortic arch syndrome secondary to a large penetrating atherosclerotic ulcer, one Takutsubo cardiomyopathy and six not further described (Supplementary Table S1). Comparison to a list of 123 cases of TAK known to the Imperial College service found 120 were identifiable in the HES, therefore sensitivity was 97.5% (95% CI 93.0, 99.5) at the time of this study.

#### Validation of diagnosis date refinement algorithm

Our initial estimate of diagnosis date was the earliest detected ICD-10 code for TAK in the HES APC. We then tested refining this date to preceding conditions and/or procedures suggesting the treatment of TAK even if not coded as such. Using data from the Imperial College service, F.P., P.L. and M.R. developed lists of TAK-suggestive conditions and procedures (Supplementary Tables S2 and S3), which were compared. The combination of list 1 (codes for aortic valve insufficiency; occlusion, stenosis, graft or aneurysm of the artery; less-specific vasculitis; chemotherapy) and attendance at an outpatient clinic for rheumatology, renal medicine or vascular surgery performed the best and is the strategy we used in phase 2 (Supplementary Fig. S1).

### Phase 2: Epidemiology study

The epidemiology study used de-identified and pseudonymised data, held in NHS England’s Secure Data Environment (SDE) service for England, made available via the BHF Data Science Centre’s CVD-COVID-UK/COVID-IMPACT Consortium (project CCU075). The consortium has ethical approval by the North East–Newcastle and North Tyneside 2 research ethics committee (REC no. 20/NE/0161).

#### Cohort demographics

All HES APC episodes and HES outpatient (OP) appointments with an ICD-10 code M31.4 in any diagnosis position were extracted for individuals with valid NHS numbers. Age at diagnosis was calculated based on episode start date and month and year of birth from the HES APC record or age at attendance from the HES OP record, whichever was the earliest. Those <2 years old were excluded, as diagnostic codes at this age were thought to be clinically unreliable [10–12]. Ethnicity was extracted from the HES APC or HES OP and categorised as Asian or Asian British, Black or Black British, Mixed, White, Other and Unknown. Where patients had more than one recorded ethnicity, the most frequent was used (and most recent if two occurred equally). Index of multiple deprivation (IMD; England’s official measure of deprivation) quintiles at the date of diagnosis and point prevalence date were based on geographic area [lower layer super output area (LSOA)] from the HES APC and HES OP [13]. There were no missing age and gender data. We describe how we handled missing LSOA data in the supplementary material.

#### Statistical methods

Crude annual incidence of TAK was calculated for 1 April 2006 to 31 March 2023 using Office for National Statistics (ONS) mid-year population estimates as the denominator [14]. People diagnosed with TAK alive and resident in England on 1 July 2022 were included when calculating crude prevalence, using the mid-2022 population denominator. Two-sided exact Poisson CIs (95%) were calculated for both crude incidence and prevalence.

To investigate whether incidence differed during the COVID-19 pandemic we calculated a rate ratio for 2020–2021 compared with all other years using Poisson regression with the log link function, adjusting for calendar year. To explore the proportion of people with TAK who were coded as such in their outpatient records, and whether this differed because of the COVID-19 pandemic, we compared how many received a diagnostic code for TAK in the HES OP before *vs* after 11 March 2020.

For each demographic characteristic, unadjusted rate ratios with 95% CIs were calculated using Poisson regression with the log link function. Adjusted rate ratios (age and gender) for IMD and Government Office Region were also produced using data published by aggregating ONS population estimates [14]. As the population breakdown of IMD for 2022 was unavailable, the 2021 estimates were reused. Rate ratios for ethnicity were separately adjusted for age and gender using ONS data from the 2011 census [15] for up to 2015 and the 2021 census [16] for 2016 onwards.

Case counts have been rounded to the nearest multiple of 5 (or suppressed if <10) to comply with NHS England’s disclosure control protocol. That is except for the Consolidated Standards of Reporting Trials flow diagram, where exact values were permitted for transparency by the NHS England Output Checker. Crude estimates presented were calculated using the rounded counts. To preserve as much data as possible without risking disclosure of personally identifiable information, unadjusted rate ratios were calculated using rounded counts if <50, otherwise unrounded counts, with the final estimates rounded to one decimal place. For adjusted rate ratios, as the level of complexity prevents any reverse engineering of case counts, unrounded counts were used. Thus the uncertainty introduced by rounding elsewhere is accounted for in the adjusted model.

Cohort creation was conducted by M.M. using PySpark in Databricks (Apache Spark 3.3.0; Apache Software Foundation, Wilmington, DE, USA) in NHS England’s SDE. Analyses were conducted by S.L. using R version 4.2.3 (R Foundation for Statistical Computing, Vienna, Austria) including the poisson.test() and glm() functions [17]. The study protocol, reproducible code and codelist are available at https://github.com/BHFDSC/CCU075_01 [18].

## Results

### Demographics

There were 1792 cases of TAK identified from 1997 onwards, of which 1235 were incident in England from 1 April 2006 to 31 March 2023 (Supplementary Fig. S2). Demographic data are shown in Table 1. For 33% of individuals, diagnosis date corresponded to the first appearance of the M31.4 code in either the HES APC or HES OP. Overall, 46% of participants had diagnosis date refinement of ≥1 year; this increased from 42% of those receiving their first TAK code prior to 11 March 2020 to 55% of those receiving their first TAK code on or after the pandemic was declared (Supplementary Table S4).

**Table 1:** Demographics of incident cases of Takayasu arteritis in England 2006–2007 to 2022–2023.

Characteristics	Cases^a^, *n* (%)	Age at diagnosis, years, median (IQR)
Overall	1235 (100)	53 (34–66.5)
Gender		
Female	910 (74)	53 (34–65)
Male	325 (26)	54.5 (35–69)
Ethnicity		
Asian	140 (11)	35 (27–48.5)
Black	40 (3)	32 (19–48.5)
Mixed	10 (1)	47 (26.5–61)
White	910 (74)	57 (40–69)
Other	40 (3)	37 (23.75–53.25)
Unknown	95 (8)	53 (37–64)
IMD		
1—most deprived	265 (21)	41 (26–58)
2	240 (19)	51 (34–65)
3	245 (20)	54 (36–65)
4	215 (17)	56 (35.5–69)
5—least deprived	270 (22)	61 (47.25–71)
Region		
East Midlands	90 (7)	50 (33.5–67.5)
East of England	175 (14)	57 (40–68)
London	210 (17)	43.5 (28–58)
North East	60 (5)	50 (31–64)
North West	160 (13)	55 (32–65)
South East	190 (15)	61 (44–72)
South West	130 (11)	58.5 (46.75–67.25)
West Midlands	90 (7)	46 (30–59.5)
Yorkshire and The Humber	130 (11)	54 (35–65)

IQR: interquartile range.

a Cases rounded to nearest 5 to comply with disclosure control protocol.

### Incidence

**Figure 1:**
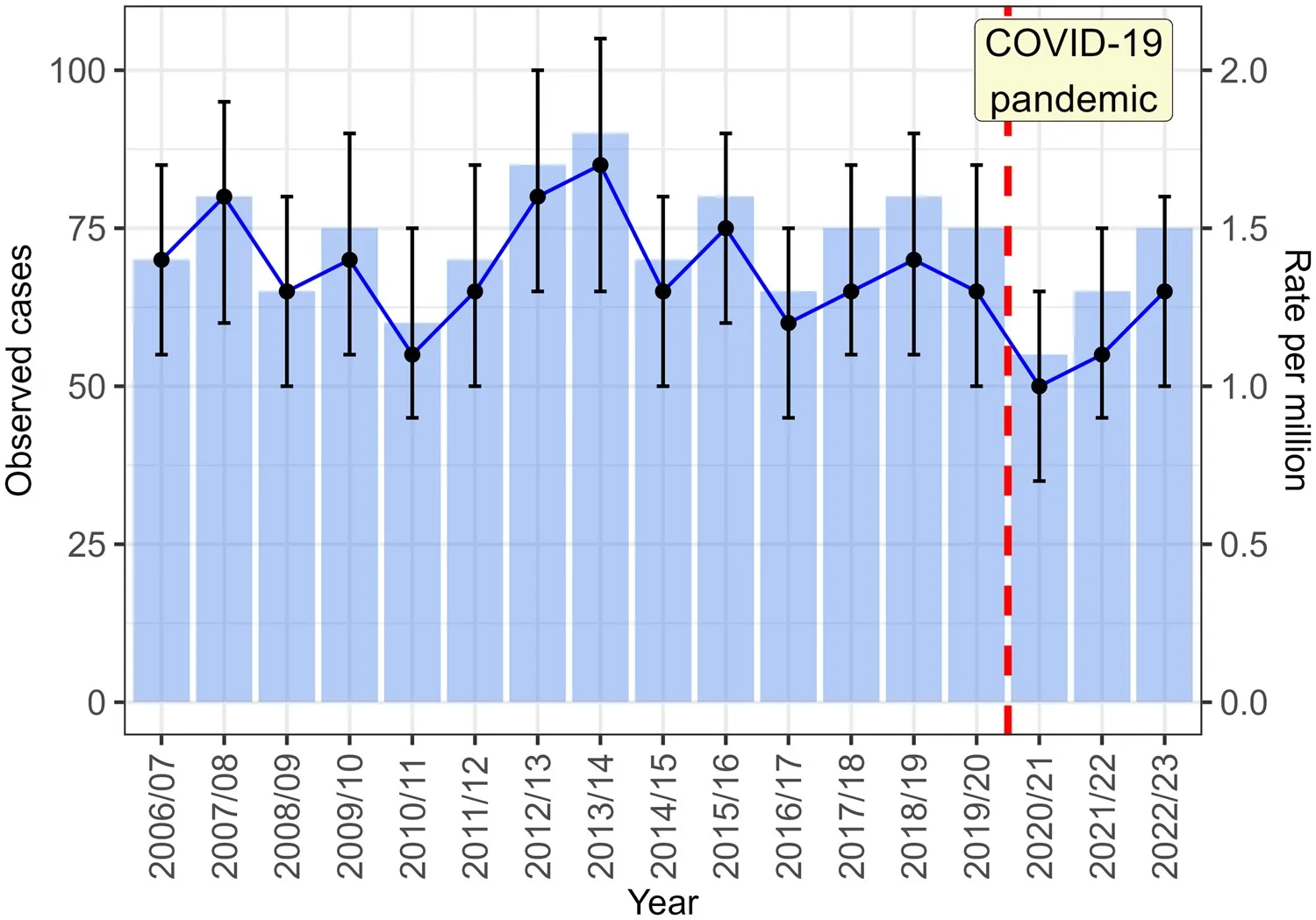
Annual incidence of Takayasu arteritis in England, with 95% Poisson CIs. Observed cases (left axis/bars) and rates (right axis/points). Cases rounded to nearest 5 to comply with disclosure control protocol.

The annual incidence ranged from 1.0 (95% CI 0.7, 1.3) to 1.7 (95% CI 1.3, 2.1) per million and was 1.3 (95% CI 1.3, 1.4) overall. While it was stable over time (*P* = 0.3), the year 2020–2021 had a lower incidence [rate ratio 0.7 (95% CI 0.6, 1.0), *P* = 0.03] compared with all other years; see Table 2 and Fig. 1]. HES years are 1 April–31 March, so the 2020–2021 year included the first and second national COVID-19 lockdowns.

**Table 2:** Annual incidence of Takayasu arteritis in England 2006–2007 to 2022–2023.

Year	Cases^a^, *n*	Population^b^, *n*	Crude incidence (95% CI)^c^
2006–2007	70	50 965 186	1.4 (1.1, 1.7)
2007–2008	80	51 381 093	1.6 (1.2, 1.9)
2008–2009	65	51 815 853	1.3 (1.0, 1.6)
2009–2010	75	52 196 381	1.4 (1.1, 1.8)
2010–2011	60	52 642 452	1.1 (0.9, 1.5)
2011–2012	70	53 107 169	1.3 (1.0, 1.7)
2012–2013	85	53 506 812	1.6 (1.3, 2.0)
2013–2014	90	53 918 686	1.7 (1.3, 2.1)
2014–2015	70	54 370 319	1.3 (1.0, 1.6)
2015–2016	80	54 808 676	1.5 (1.2, 1.8)
2016–2017	65	55 289 034	1.2 (0.9, 1.5)
2017–2018	75	55 619 548	1.3 (1.1, 1.7)
2018–2019	80	55 924 528	1.4 (1.1, 1.8)
2019–2020	75	56 230 056	1.3 (1.0, 1.7)
2020–2021	55	56 325 961	1.0 (0.7, 1.3)
2021–2022	65	56 554 891	1.1 (0.9, 1.5)
2022–2023	75	57 106 398	1.3 (1.0, 1.6)
Total	1235	921 763 043	1.3 (1.3, 1.4)

a Cases rounded to nearest 5 to comply with disclosure control protocol.

b Mid-year population estimates [14].

c Incident cases per million with two-tailed 95% Poisson CI.

The incidence of TAK was significantly higher in women than men, with a rate ratio of 2.7 (95% CI 2.4, 3.1). The incidence of TAK was lower at extremes of age and was highest in the 55–69 and 70–84 years age categories (Table 3). The median age in years of incident cases was consistent over time (Supplementary Fig. S3). Using the 16–39 years age group as a comparator, the incidence of TAK was 2.0 (95% CI 1.7, 2.3) times higher in those 55–69 years and 2.0 (95% CI 1.7, 2.4) times higher in those 70–84 years.

**Table 3:** Overall incidence of Takayasu arteritis in England 2006–2007 to 2022–2023.

Characteristics	Cases^a^, *n* (%)	Population, person-years^b^	Crude incidence (95% CI)^c^	Crude incidence rate ratio (95% CI)^d^	Adjusted incidence rate ratio (95% CI)^d^^,e^
Overall	1235 (100)	921 763 043	1.3 (1.3, 1.4)		
Gender					
Female	910 (74)	469 260 686	1.9 (1.8, 2.1)	2.7 (2.4, 3.1)	2.6 (2.3, 3)
Male	325 (26)	452 502 357	0.7 (0.6, 0.8)	1	1
Age group (years)					
0–15	65 (5)	173 860 290	0.4 (0.3, 0.5)	0.3 (0.3, 0.4)	0.4 (0.3, 0.5)
16–39	325 (26)	291 516 886	1.1 (1.0, 1.2)	1	1
40–54	255 (21)	188 919 934	1.3 (1.2, 1.5)	1.2 (1.0, 1.4)	1.2 (1.0, 1.4)
55–69	340 (28)	153 900 256	2.2 (2.0, 2.5)	2.0 (1.7, 2.3)	2.0 (1.7, 2.3)
70–84	210 (17)	92 290 470	2.3 (2.0, 2.6)	2.0 (1.7, 2.4)	2.0 (1.7, 2.3)
≥85	35 (3)	21 275 207	1.6 (1.1, 2.3)	1.5 (1.0, 2.1)	1.4 (1.0, 1.9)
Ethnicity					
Asian	140 (11)	79 079 641	1.8 (1.5, 2.1)	1.5 (1.2, 1.8)	1.9 (1.6, 2.2)
Black	40 (3)	34 988 125	1.1 (0.8, 1.6)	1.0 (0.7, 1.3)	1.2 (0.9, 1.7)
Mixed	10 (1)	23 510 792	0.4 (0.2, 0.8)	0.4 (0.2, 0.6)	1.0 (0.6, 1.6)
White	910 (74)	770 162 737	1.2 (1.1, 1.3)	1	1
Other	40 (3)	14 021 748	2.9 (2.0, 3.9)	2.4 (1.7, 3.3)	3.4 (2.4, 4.5)
Unknown	95 (8)				
IMD					
1–most deprived	265 (21)	184 652 849	1.4 (1.3, 1.6)	1	1
2	240 (19)	187 920 743	1.3 (1.1, 1.4)	0.9 (0.8, 1.1)	0.9 (0.7, 1.0)
3	245 (20)	185 846 260	1.3 (1.2, 1.5)	0.9 (0.8, 1.1)	0.9 (0.7, 1.0)
4	215 (17)	182 894 529	1.2 (1.0, 1.3)	0.8 (0.7, 1.0)	0.7 (0.6, 0.9)
5–least deprived	270 (22)	180 434 196	1.5 (1.3, 1.7)	1.1 (0.9, 1.3)	0.9 (0.8, 1.1)
Region					
North East	60 (5)	44 343 300	1.4 (1.1, 1.7)	0.9 (0.7, 1.2)	0.9 (0.7, 1.2)
North West	160 (13)	121 869 783	1.3 (1.1, 1.5)	0.9 (0.7, 1.1)	0.8 (0.7, 1.0)
Yorkshire and The Humber	130 (11)	90 882 121	1.4 (1.2, 1.7)	1.0 (0.8, 1.2)	0.9 (0.7, 1.1)
East Midlands	90 (7)	79 069 366	1.1 (0.9, 1.4)	0.8 (0.6, 1.0)	0.7 (0.6, 0.9)
West Midlands	90 (7)	97 244 072	0.9 (0.7, 1.1)	0.6 (0.5, 0.8)	0.6 (0.5, 0.7)
East of England	175 (14)	102 334 136	1.7 (1.5, 2.0)	1.2 (1.0, 1.4)	1.1 (0.9, 1.3)
London	210 (17)	143 059 361	1.5 (1.3, 1.7)	1	1
South East	190 (15)	150 577 955	1.3 (1.1, 1.5)	0.9 (0.7, 1.1)	0.8 (0.7, 1.0)
South West	130 (11)	92 382 949	1.4 (1.2, 1.7)	0.9 (0.8, 1.2)	0.9 (0.7, 1.1)

a Cases rounded to nearest 5 to comply with disclosure control protocol.

b Based on Office for National Statistics population estimates and BHF Data Science Centre curated assets.

c Incident cases per million with two-tailed 95% Poisson CI.

d CIs calculated using the standard error of the log incidence rate ratio.

e Incidence rate ratios adjusted for age and gender. Age-specific rates were adjusted by gender only. Gender-specific rates were adjusted by age only.

Incidence was highest in the Other and Asian/Asian British categories: 2.9/million (95% CI 2.0, 3.9) and 1.8/million (95% CI 1.5, 2.1), respectively. Adjustment for age and gender increased the incidence rate ratios and the associations persisted in sensitivity analyses where Unknown ethnicities were assumed to be White rather than excluded (Supplementary Table S5). The adjusted rate ratio for Asian/Asian British compared with White was 1.9 (95% CI 1.6, 2.2) and for Other compared with White was 3.4 (95% CI 2.4, 4.5) (Table 3).

To explore this further, we conducted post hoc analyses with binary ethnicity (White *vs* non-White) modelled as an interaction with age group (Supplementary Tables S6 and S7). In non-White individuals, the incidence rate ratio was relatively similar across age groups (excluding ≥85 years, where small numbers have most likely driven a spuriously high result). This contrasts with White individuals, where there were 2-fold increased estimates in the 55–84 years age groups compared with 16–39 years. Across the various models, age- and sex-specific incidence rate ratios remained similar (Supplementary Table S8).

The incidence of TAK was highest in the East of England [1.7/million (95% CI 1.5, 2.0)] and London [1.5/million (95% CI 1.3, 1.7)]. Compared with London, the incidence of TAK was markedly lower in the West Midlands [0.6/million (95% CI 0.5, 0.8)]. This persisted after adjustment for age and gender. While there was variation in incidence across the IMD quintiles, there was no clear trend.

### Prevalence

The overall prevalence of TAK in England in mid-2022 was 22.5/million (95% CI 21.3, 23.8; Table 4), based on 1285 individuals. The prevalence in women was 34.3/million and in men 10.2/million, giving a rate ratio of 3.3 (95% CI 2.9, 3.8). Prevalence was lowest in those <15 years of age, then increased through the age categories to peak between age 70 and 84, before decreasing slightly in the ≥85 age category.

**Table:** **Table 4** Mid-2022 point prevalence of Takayasu arteritis per million.

Characteristics	Cases^a^, *n* (%)	Population^b^, *n*	Crude prevalence (95% CI)^c^	Crude prevalence rate ratio (95% CI)^d^	Adjusted prevalence rate ratio (95% CI)^d^^,^^e^
Overall	1285 (100)	57 106 398	22.5 (21.3, 23.8)		
Gender					
Female	1000 (78)	29 123 108	34.3 (32.2, 36.5)	3.3 (2.9, 3.8)	3.2 (2.8, 3.6)
Male	285 (22)	27 983 290	10.2 (9.0, 11.4)	1	1
Age group (years)					
0–15	30 (2)	10 575 320	2.8 (1.9, 4.0)	0.2 (0.1, 0.3)	0.3 (0.2, 0.4)
16–39	235 (18)	17 634 431	13.3 (11.7, 15.1)	1	1
40–54	290 (23)	11 031 939	26.3 (23.3, 29.5)	2.0 (1.7, 2.4)	2.0 (1.7, 2.4)
55–69	350 (27)	10 060 648	34.8 (31.2, 38.6)	2.6 (2.2, 3.1)	2.6 (2.2, 3.1)
70–84	325 (25)	6 379 189	50.9 (45.6, 56.8)	3.9 (3.3, 4.6)	3.8 (3.2, 4.5)
≥85	55 (4)	1 424 871	38.6 (29.1, 50.2)	2.9 (2.2, 3.9)	2.8 (2.0, 3.6)
Ethnicity					
Asian	165 (12)	5 426 315	30.4 (25.9, 35.4)	1.5 (1.3, 1.7)	2.1 (1.8, 2.5)
Black	50 (4)	2 381 620	21 (15.6, 27.7)	1.0 (0.8, 1.4)	1.4 (1.0, 1.8)
Mixed	15 (1)	1 669 140	9.0 (5.0, 14.8)	0.4 (0.3, 0.7)	1.0 (0.6, 1.6)
White	930 (72)	45 783 290	20.3 (19.0, 21.7)	1	1
Other	40 (3)	1 229 125	32.5 (23.2, 44.3)	1.6 (1.2, 2.2)	2.4 (1.7, 3.2)
Unknown	90 (7)				
IMD					
1–most deprived	260 (20)	11 295 065	23.0 (20.3, 26.0)	1	1
2	235 (18)	11 626 962	20.2 (17.7, 23.0)	0.9 (0.7, 1.0)	0.8 (0.7, 1.0)
3	260 (20)	11 533 486	22.5 (19.9, 25.5)	1.0 (0.8, 1.2)	0.8 (0.7, 1.0)
4	240 (19)	11 254 837	21.3 (18.7, 24.2)	0.9 (0.8, 1.1)	0.8 (0.6, 0.9)
5–least deprived	290 (23)	11 027 739	26.3 (23.4, 29.5)	1.1 (1.0, 1.3)	0.9 (0.8, 1.1)
Region					
North East	60 (5)	2 683 040	22.4 (17.1, 28.8)	0.9 (0.6, 1.1)	0.8 (0.6, 1.0)
North West	165 (13)	7 516 113	22.0 (18.7, 25.6)	0.8 (0.7, 1.0)	0.8 (0.6, 0.9)
Yorkshire and The Humber	135 (11)	5 541 262	24.4 (20.4, 28.8)	0.9 (0.7, 1.1)	0.8 (0.7, 1.0)
East Midlands	95 (7)	4 934 939	19.3 (15.6, 23.5)	0.7 (0.6, 0.9)	0.6 (0.5, 0.8)
West Midlands	100 (8)	6 021 653	16.6 (13.5, 20.2)	0.6 (0.5, 0.8)	0.6 (0.5, 0.7)
East of England	170 (13)	6 398 497	26.6 (22.7, 30.9)	1.0 (0.8, 1.2)	0.9 (0.7, 1.1)
London	235 (18)	8 866 180	26.5 (23.2, 30.1)	1	1
South East	200 (16)	9 379 833	21.3 (18.5, 24.5)	0.8 (0.7, 1.0)	0.7 (0.6, 0.9)
South West	125 (19)	5 764 881	21.7 (18.0, 25.8)	0.8 (0.7, 1.0)	0.7 (0.6, 0.9)

a Cases rounded to nearest 5 to comply with disclosure control protocol.

b Based on Office for National Statistics population estimates and BHF Data Science Centre curated assets [14].

c Prevalent cases per million with two-tailed 95% Poisson CI.

d CIs calculated using the standard error of the log prevalence rate ratio.

e Prevalence rate ratios adjusted for age and gender. Age-specific rates were adjusted by gender only. Gender-specific rates were adjusted by age only.

The highest prevalence was observed in Asian/Asian British [rate ratio 1.5 (95% CI 1.3, 1.7) compared with White] and Other ethnic categories [rate ratio 1.6 (95% CI 1.2, 2.2) compared with White]. As for incidence, adjustment for age and gender increased these prevalence rate ratios and the associations persisted in sensitivity analyses (Supplementary Table S9). However, in contrast to incidence, a post hoc analysis with binary ethnicity modelled as an interaction with age group (Supplementary Table S10) showed similar age-specific prevalence rate ratios for White and non-White individuals.

**Figure 2:**
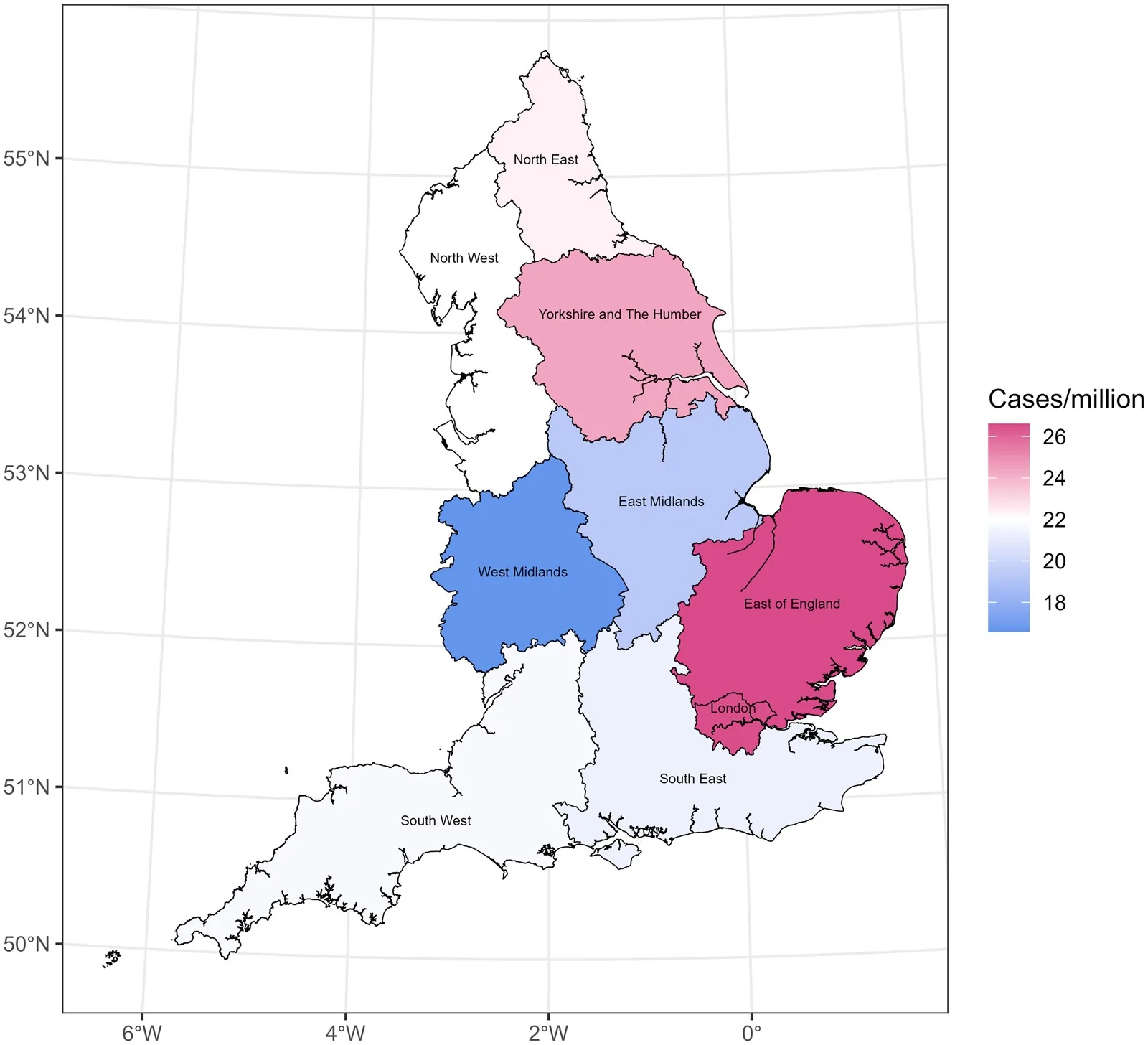
Crude mid-2022 point prevalence rate per million of Takayasu arteritis by English Government Office Region.

The prevalence of TAK, shown in Fig. 2, was highest in the East of England [26.6/million (95% CI 22.7, 30.9)] and London [26.5/million (95% CI 23.2, 30.1)]. Compared with London, the prevalence of TAK was markedly lower in the West Midlands [0.6/million (95% CI 0.5, 0.8)] and East Midlands [0.7/million (95% CI 0.6, 0.9)]. Adjustment for age and gender decreased the prevalence rate ratios for most regions. There was no clear association with deprivation. Further exploration of the intersectionality of region, ethnicity and deprivation of prevalent cases is included in Supplementary Table S10 and Supplementary Figs. S4–S7.

We have demonstrated all crude incidence and prevalence estimates are robust to the uncertainty introduced by rounding of case counts in sensitivity analyses (Supplementary Table S11).

### HES APC and HES OP recording habits pre- and post-COVID-19

Incident cases of TAK in England from 1 April 2006 to 31 March 2023 had been coded by 173 unique NHS trusts, 23 of whom had coded diagnoses in the HES OP (13%). Considering only incident cases prior to 11 March 2020, 11% of NHS trusts had coded TAK diagnoses in the HES OP (see Supplementary Table S12). Of the incident cases diagnosed on or after 11 March 2020, 10% of NHS trusts had coded TAK diagnoses in the HES OP. Fourteen NHS trusts had prevalent caseloads of at least 20 cases.

## Discussion

The overall annual incidence of TAK in England between 2006 and 2023 was 1.3/million (95% CI 1.3, 1.4), with an almost three times higher incidence in females than males. This aligns with the findings of our recent worldwide systematic review of TAK epidemiology where the overall pooled incidence was 1.1 (95% CI 0.7, 1.8) and estimates were significantly higher among women than men [7]. Our estimate of prevalence was 22.5/million in mid-2022 (95% CI 21.3, 23.8), which is very similar to the prevalence found by a large population-based study in Norway in 2017 [22/million (95% CI 17, 29)] [19]. Prevalence is accepted to be higher in Asian populations, and our findings fit this pattern, being lower than the prevalence reported in Asian populations (28.3/million in South Korea 2017 [20], 32.5/million in South Korea in 2022 [21] and 33/million in Turkey in 2016 [22]).

The year April 2020–March 2021 had a notably low incidence, corresponding to most of the first and second national COVID-19 lockdowns. It is likely this represents fewer hospital admissions for non-COVID illnesses as shown across multiple other conditions in both the UK and USA [23, 24] and there is evidence from cancers that not only were there fewer admissions, but also fewer diagnoses occurred [25]. It is also possible more diagnoses and treatment of TAK occurred in outpatients, where minimal TAK coding occurs.

Of note, our study’s incidence based on clinical diagnoses was highest in 55–84-year-olds. Absolute age cut-offs included in epidemiological classification criteria complicate the literature around age at incidence in TAK, with older criteria requiring an age <40 years at onset (ACR 1990 [26] and Ishikawa 1988 [27]) and the newest ACR/EULAR criteria published in 2022 including an absolute age requirement ≤60 years [28]. Our findings fit with other population-based studies in the literature that did not use an age cut-off in their classification of TAK cases. The previous UK epidemiology study using a large database of general practitioner records from 2000 to 2005 found an average age at diagnosis of 51 years [4]. It is recognised that age at onset is younger in Asian than White people [19], yet two whole-nation population-based studies in South Korea report average ages at diagnosis of 46 years in cases diagnosed in 2008–2012 and 46.7 in cases diagnosed in 2010–2018 [20, 21]. The earlier of these studies included confirmed cases only—confirmed by a medical panel and meeting the 1990 ACR criteria except for age. In keeping with finding diagnoses at older ages among White individuals, we found our overall age findings were driven by statistically significant increases in the older incidence among the White ethnic group but not the non-White group (Supplementary Table S6 and S7).

The older age at diagnosis in our study may represent genuine disease onset later in life, perhaps with increased diagnoses due to modern imaging techniques, or it could be due to the acknowledged delay in diagnosis of this rare, insidious onset disease [29, 30], or even misclassification of large vessel GCA. Plausible clinical scenarios were illustrated in the ‘gold standard’ cohort of TAK patients from Imperial College. In this cohort, there were some diagnosed with active disease in their 50s–60s whose pattern of arterial injury was consistent with TAK, and some diagnosed in their 50s–60 s with burnt-out disease with arterial damage identified incidentally on imaging for another reason.

We found TAK has a significantly higher incidence and prevalence in Asian/Asian British and Other ethnic groups, which has long been considered the case [31]. Considerable variation in prevalence was seen between different geographic populations in England, which was less apparent in the incidence data. Socio-economic status did not have a marked effect on incidence or prevalence, but those from more deprived areas were younger at diagnosis, which could impact outcomes. Regrettably, it was not possible to explore residual confounding between combinations of regional, ethnic and IMD status, due to a lack of availability of combination denominator data.

A key strength of this study was collaboration with the Takayasu team at Imperial College London using their curated cohort of 153 people, 123 of whom were resident in England during the study and included in our analyses to develop the diagnosis date refinement algorithm and demonstrate 97.5% sensitivity of case ascertainment. Another key strength, enabled by collaboration with the NDRS, was to assess specificity in five other NHS trusts, demonstrating a positive predictive value of 80.6%. This positive predictive value is exactly in keeping with a previous meta-analysis of studies of UK hospital discharges that found the median diagnostic accuracy was 80.3% (interquartile range 63.3–94.1) [32]. While this suggests our results are an overestimate of incidence and prevalence, this is balanced against the likelihood we missed patients exclusively treated as outpatients, as only ≈13% of trusts routinely coded HES OP records.

This is the first time whole population data in England has been used to calculate the incidence and prevalence of TAK. The British Heart Foundation Data Science Centre enabled access to HES data for >57 million people. This identified a cohort of 1285 prevalent cases, ≈100 times the largest UK cohort in the previous epidemiology study, and nearly 10 times the cohort at Imperial College. The use of whole population data also reduced the risk of survivor bias seen in cross-sectional and cohort studies conducted in primary care data (where cases with rapid disease progression and early death are omitted and longer survivors are overrepresented) or selection bias seen in cases series from specialist centres and registry studies.

This study highlights the role of national specialist centres for ultrarare diseases and supports formal highly specialised commissioning for TAK. Imperial College London has informally offered a national tertiary referral service for TAK for the whole of the UK and has concentrated expertise based on a caseload of 153 cases. A recent audit of the Imperial College service found patients received lower doses of radiation, lower glucocorticoid exposure and had faster time to biologic medicine than people not seen in the service (unpublished data from service audit). A study of other types of vasculitis has also demonstrated that cohorted clinics (similar cases seen together in a clinic, enabling a systematic approach to management) and joint/parallel multidisciplinary team clinics resulted in fewer serious infections, fewer emergency hospital admissions and lower mortality [33].

TAK is an exemplar for many rare rheumatic diseases where we typically lack contemporary, robust epidemiological data to guide healthcare planning, including high-cost treatments, pandemic planning/mitigation, specialised clinical knowledge and resources. This study shows the power of England’s large population and universal health system to enable epidemiological research on ultrarare conditions. Policymakers could improve the accuracy of future studies by mandating diagnostic coding of outpatients. Approximately 85% of hospital-based activity in the UK (excluding accident and emergency) is made up of outpatient appointments [34], and yet data are not routinely collected about outpatient diagnoses, unlike inpatient diagnoses. Clinicians can improve the accuracy of future studies by writing notes that follow national coding rules and include the diagnosis ‘Takayasu arteritis’, which has a specific ICD-10 code. If clinicians only record ‘vasculitis’ or ‘large vessel vasculitis’, this will receive a non-specific code such as ‘arteritis unspecified’ and not be identified as TAK. Digital transformation should further improve the quality of the data over time.

## Supplementary Material

rkag078_Supplementary_Data

## Data Availability

The North East–Newcastle and North Tyneside 2 research ethics committee provided ethical approval for the CVD-COVID-UK/COVID-IMPACT research program (REC no. 20/NE/0161) to access, within secure trusted research environments, unconsented, whole-population, de-identified data from electronic health records collected as part of patients’ routine healthcare. The data used in this study are available in NHS England’s SDE service for England, but as restrictions apply, they are not publicly available (https://digital.nhs.uk/services/secure-data-environment-service). The CVD-COVID-UK/COVID-IMPACT program, led by the BHF Data Science Centre (https://bhfdatasciencecentre.org/), received approval to access data in NHS England’s SDE service for England from the Independent Group Advising on the Release of Data (https://digital.nhs.uk/about-nhs-digital/corporate-information-and-documents/independent-group-advising-on-the-release-of-data) via an application made in the Data Access Request Service online system (ref. DARS-NIC-381078-Y9C5K; https://digital.nhs.uk/services/data-access-request-service-dars/dars-products-and-services). The CVD-COVID-UK/COVID-IMPACT Approvals & Oversight Board (https://bhfdatasciencecentre.org/areas/cvd-covid-uk-covid-impact/) subsequently granted approval to this project to access the data within NHS England’s SDE service for England. The de-identified data used in this study were made available to accredited researchers only. Those wishing to gain access to the data should contact bhfdsc@hdruk.ac.uk.
