# Supplementary material for: Incidence and prevalence of Takayasu arteritis in England before and after the COVID-19 pandemic: results from RECORDER
Source: Rheumatol Adv Pract. 2026 Jul 24;10(3):rkag078. doi: 10.1093/rap/rkag078 (PMC13493044; doi:10.1093/rap/rkag078)
Supplement: Supplementary file 1 [file rkag078_supplementary_data.zip › 20260811_Supplementary_materials.docx]

Supplementary material

Contents

[Case validation and date refinement	3](#_Toc237335920)

[Case validation and date refinement methods	3](#_Toc237335921)

[Details of validation of case ascertainment and date of diagnosis	3](#_Toc237335922)

[Cohort identification	3](#_Toc237335923)

[Stage 1: Initial validation	4](#_Toc237335924)

[Stage 2: Diagnosis date refinement strategy	4](#_Toc237335925)

[Stage 3: Evaluation of strategy	4](#_Toc237335926)

[Stage 4: Further refinement using HES Outpatient data	5](#_Toc237335927)

[Case validation results	5](#_Toc237335928)

[**Supplementary Table S1:** Initial validation of cases of Takayasu arteritis	5](#_Toc237335929)

[Date refinement methods	6](#_Toc237335930)

[**Supplementary Table S2:** Code lists tested for refinement of estimated diagnosis date	6](#_Toc237335931)

[**Supplementary Table S3:** Code lists for diagnosis date refinement	10](#_Toc237335932)

[**Supplementary Table S4:** Summary of the extent of diagnosis date refinement for incident cases of Takayasu arteritis in England 2006/07 to 2022/23	10](#_Toc237335933)

[**Supplementary Figure S1:** Comparison of diagnosis date adjustment strategies	11](#_Toc237335934)

[Epidemiology of Takayasu Arteritis	13](#_Toc237335935)

[Additional methods	13](#_Toc237335936)

[Ascertainment of IMD from available data – additional details	13](#_Toc237335937)

[Additional results	13](#_Toc237335938)

[**Supplementary Table S5:** Overall incidence of Takayasu arteritis (per million people) by ethnicity: sensitivity analysis assuming all individuals with unknown ethnicity are White	13](#_Toc237335939)

[**Supplementary Table S6:** Overall incidence of Takayasu arteritis (per million people): post-hoc analysis where age and ethnicity are modelled as an interaction using Poisson regression	14](#_Toc237335940)

[**Supplementary Table S7:** Overall incidence of Takayasu arteritis (per million people): sensitivity analysis where age and ethnicity are modelled as an interaction using the negative binomial distribution	14](#_Toc237335941)

[**Supplementary Table S8:** Overall incidence of Takayasu arteritis (per million people): sensitivity analysis of age- and sex-specific incidence rate ratios derived from multivariable models with ethnicity, IMD, and region	16](#_Toc237335942)

[**Supplementary Table S9:** Overall prevalence of Takayasu arteritis (per million people) by ethnicity: sensitivity analysis assuming all individuals with unknown ethnicity are White	17](#_Toc237335943)

[**Supplementary Table S10:** Overall prevalence of Takayasu arteritis (per million people): post-hoc analysis where age and ethnicity are modelled as an interaction using Poisson regression	17](#_Toc237335944)

[**Supplementary Table S11:** Sensitivity analysis of crude incidence and prevalence rates to account for the uncertainty introduced by rounding of case counts.	18](#_Toc237335945)

[**Supplementary Table S12:** Summary of NHS providers’ TAK coding habits in the incident cohort.	22](#_Toc237335946)

[**Supplementary Figure S2:** Flow diagram of cohort construction from HES Admitted Patient Care (APC) and HES Outpatients (OP) data	23](#_Toc237335947)

[**Supplementary Figure S3:** Median age in years of incident cases of Takayasu arteritis (IQR)	24](#_Toc237335948)

[**Supplementary Figure S4:** Region of residence of prevalent cases of Takayasu arteritis: white versus other/unknown ethnicity	25](#_Toc237335949)

[**Supplementary Figure S5:** Ethnicity of prevalent cases of Takayasu arteritis: London residents versus other	25](#_Toc237335950)

[**Supplementary Figure S6:** IMD of prevalent cases of Takayasu arteritis by region (IMD 1 = most deprived; 5 = least)	26](#_Toc237335951)

[**Supplementary Figure S7:** IMD of prevalent cases of Takayasu arteritis by ethnicity (IMD 1 = most deprived; 5 = least)	27](#_Toc237335952)

# Case validation and date refinement

## Case validation and date refinement methods

### Details of validation of case ascertainment and date of diagnosis

Validation was conducted by the RECORDER collaboration between the University of Nottingham and NDRS. We used NDRS’ legal and ethical permissions under Section 254 of the Health and Social Care Act 2012, under the National Disease Registries Directions 2021 (Public Health England, 2015), to access Hospital Episode Statistics (HES) data. HES contains details of all episodes of admitted patient care (APC) and outpatient (OP) appointments at NHS hospitals in England. HES APC data have been collected since 1997/98, recording the main diagnosis for each admission and all prevalent diagnoses coded according to ICD-10. HES OP data have been collected since 2003/4 and include every OP appointment in England, recording the hospital specialty, and in a small minority of appointments, the main diagnosis.

### Cohort identification

All HES APC episodes with ICD-10 code M31.4 for “Aortic arch syndrome [Takayasu]” in any diagnosis position were extracted. Data were restricted to cases with valid NHS numbers. Date of birth, gender, post code, and, where relevant, date of death, were linked by means of the NHS Personal Demographics Service. Using the earliest TAK code as the initial estimated diagnosis date, those diagnosed aged less than 2 were excluded from the cohort as TAK is very rare in this age group, typically presenting in early adulthood, and diagnostic codes at this age were clinically thought to be unreliable (Brunner et al., 2010; Eleftheriou et al., 2015; Sahin et al., 2019). All available HES APC episodes were extracted for everyone in the cohort, to allow further refinement of the date of diagnosis as described below.

HES OP records with an ICD-10 code for TAK were also extracted and individuals were added to the cohort if they had an OP diagnostic code for TAK and at least one HES APC record. Date of birth and gender were obtained from the NHS Personal Demographics Service.

Validation

### Stage 1: Initial validation

NDRS’ data sharing agreements with NHS Trusts in England enabled us to confirm the diagnosis in a sample of records to validate the methodology. HES APC episodes between 2003 and 2017 with the ICD-10 diagnostic code for TAK (M31.4), as either the primary or co-morbid diagnosis, were validated in 5 hospital trusts (n=62), with a positive predictive value of 80.6% (68.6-89.6) (Supplementary Table S1). Comparison to the list of 123 cases of TAK known to the Imperial College vasculitis service and who were resident in England found 120 were identifiable in HES. Sensitivity was therefore 97.5 % (95% CI 93.0-99.5%) at the time of this study, but it is important to remember that sensitivity increases with length of time after diagnosis as there is more opportunity for subsequent coded hospital admissions to occur.

### Stage 2: Diagnosis date refinement strategy

People with TAK are often diagnosed and treated in outpatients and may not have a hospital admission with a diagnostic code for TAK for some time after their diagnosis. Our initial estimate of diagnosis date was the earliest detected ICD-10 code for TAK in HES APC. Refinement of this date was based on the premise that some preceding conditions and/or procedures suggest the treatment of TAK even if this is not coded as TAK. Using data from 120 people with TAK known to the Imperial College Takayasu service, FP, PL and MR developed three lists of TAK-suggestive conditions and procedures (Supplementary tables S2 & S3) including “Aortic valve insufficiency”, “Occlusion and stenosis of carotid artery” and “Arteritis unspecified”.

### Stage 3: Evaluation of strategy

Using the Imperial college hospital sample (n=120), for whom we had both the formal date of TAK diagnosis and HES APC data giving our initial estimated date of TAK diagnosis, we tested different refinement methods based on the three code lists. Based on Median Absolute Error (MedAE) in days between the revised estimated date of diagnosis and the accurate Imperial one, list 1 alone performed best (MedAE=314), outperforming lists 1 and 2 combined and 1, 2 and 3 combined.

### Stage 4: Further refinement using HES Outpatient data

For an individual with a future diagnostic code for TAK, attendance at an OP clinic for any of rheumatology, renal medicine or vascular surgery, suggests that they already have TAK. Therefore, if any such earlier OP appointment was found, the estimated diagnosis date was revised accordingly. The combination of list 1 and OP data improved accuracy further and is the strategy we recommend (MedAE=291, Supplementary figure S1).

## Case validation results

### **Supplementary Table S1:** Initial validation of cases of Takayasu arteritis

|  | No of cases from HES | Notes available | No of cases confirmed | PPV (95% CI)^1^ |
| --- | --- | --- | --- | --- |
| Bristol | 15 | 12 | 10 | 83.3% |
| Southampton | 20 | 14 | 10 | 71.4% |
| Leeds | 14 | 13 | 11 | 84.6% |
| Nottingham | 12 | 12 | 11 | 91.7% |
| Newcastle | 12 | 11 | 8 | 72.7% |
| **Combined** | **73** | **62** | **50** | **80.6% (68.6-89.6)** |

^1^ Binomial exact confidence interval

The cases which had sufficient information in the notes but in which the diagnosis was not TAK were: 2 unclassified vasculitis, 1 SAPHO, 1 former Kawasaki disease, 1 aortic arch syndrome secondary to a large penetrating atherosclerotic ulcer, 1 Takutsubo cardiomyopathy, and 6 not further described.

## Date refinement methods

### **Supplementary Table S2:** Code lists tested for refinement of estimated diagnosis date

| List# | Code | Type | Description |
| --- | --- | --- | --- |
| 1 | I351 | ICD-10 | Aortic (valve) insufficiency |
| 1 | I652 | ICD-10 | Occlusion and stenosis of carotid artery |
| 1 | I712 | ICD-10 | Thoracic aortic aneurysm, without mention of rupture |
| 1 | I771 | ICD-10 | Stricture of artery |
| 1 | I776 | ICD-10 | Arteritis, unspecified |
| 1 | M316 | ICD-10 | Other giant cell arteritis |
| 1 | Z512 | ICD-10 | Other chemotherapy |
| 1 | Z958 | ICD-10 | Presence of other cardiac and vascular implants and grafts |
|  |  |  |  |
| 2 | H150 | ICD-10 | Scleritis |
| 2 | I080 | ICD-10 | Disorders of both mitral and aortic valves |
| 2 | I081 | ICD-10 | Disorders of both mitral and tricuspid valves |
| 2 | I120 | ICD-10 | Hypertensive renal disease with renal failure |
| 2 | I150 | ICD-10 | Renovascular hypertension |
| 2 | I270 | ICD-10 | Primary pulmonary hypertension |
| 2 | I272 | ICD-10 | Other secondary pulmonary hypertension |
| 2 | I288 | ICD-10 | Other specified diseases of pulmonary vessels |
| 2 | I340 | ICD-10 | Mitral (valve) insufficiency |
| 2 | I350 | ICD-10 | Aortic (valve) stenosis |
| 2 | I352 | ICD-10 | Aortic (valve) stenosis with insufficiency |
| 2 | I358 | ICD-10 | Other aortic valve disorders |
| 2 | I501 | ICD-10 | Left ventricular failure |
| 2 | I517 | ICD-10 | Cardiomegaly |
| 2 | I518 | ICD-10 | Other ill-defined heart diseases |
| 2 | I632 | ICD-10 | Cerebral infarction due to unspecified occlusion or stenosis of precerebral arteries |
| 2 | I650 | ICD-10 | Occlusion and stenosis of vertebral artery |
| 2 | I701 | ICD-10 | Atherosclerosis of renal artery |
| 2 | I702 | ICD-10 | Atherosclerosis of arteries of extremities |
| 2 | I708 | ICD-10 | Atherosclerosis of other arteries |
| 2 | I710 | ICD-10 | Dissection of aorta [any part] |
| 2 | I719 | ICD-10 | Aortic aneurysm of unspecified site, without mention of rupture |
| 2 | I728 | ICD-10 | Aneurysm and dissection of other specified arteries |
| 2 | I730 | ICD-10 | Raynaud syndrome |
| 2 | I739 | ICD-10 | Peripheral vascular disease, unspecified |
| 2 | I745 | ICD-10 | Embolism and thrombosis of iliac artery |
| 2 | I748 | ICD-10 | Embolism and thrombosis of other arteries |
| 2 | I778 | ICD-10 | Other specified disorders of arteries and arterioles |
| 2 | I779 | ICD-10 | Disorder of arteries and arterioles, unspecified |
| 2 | K551 | ICD-10 | Chronic vascular disorders of intestine |
| 2 | K558 | ICD-10 | Other vascular disorders of intestine |
| 2 | M319 | ICD-10 | Necrotizing vasculopathy, unspecified |
| 2 | N280 | ICD-10 | Ischaemia and infarction of kidney |
| 2 | Q603 | ICD-10 | Renal hypoplasia, unilateral |
| 2 | R002 | ICD-10 | Palpitations |
| 2 | R53X | ICD-10 | Malaise and fatigue |
| 2 | R634 | ICD-10 | Abnormal weight loss |
| 2 | Y434 | ICD-10 | Immunosuppressive agents |
| 2 | Z867 | ICD-10 | Personal history of diseases of the circulatory system |
| 2 | Z951 | ICD-10 | Presence of aortocoronary bypass graft |
| 2 | Z952 | ICD-10 | Presence of prosthetic heart valve |
| 2 | Z955 | ICD-10 | Presence of coronary angioplasty implant and graft |
| 2 | Z959 | ICD-10 | Presence of cardiac and vascular implant and graft, unspecified |
| 2 | K252 | OPCS-4 | Xenograft replacement of mitral valve |
| 2 | K262 | OPCS-4 | Xenograft replacement of aortic valve |
| 2 | K334 | OPCS-4 | Aortic root replacement using mechanical prosthesis |
| 2 | L191 | OPCS-4 | Replacement of aneurysmal segment of ascending aorta by anastomosis of aorta to aorta NEC |
| 2 | L264 | OPCS-4 | Aortography |
| 2 | L391 | OPCS-4 | Percutaneous transluminal angioplasty of subclavian artery |
| 2 | L394 | OPCS-4 | Arteriography of subclavian artery |
| 2 | L431 | OPCS-4 | Percutaneous transluminal angioplasty of renal artery |
| 2 | L434 | OPCS-4 | Arteriography of renal artery |
| 2 | L435 | OPCS-4 | Percutaneous transluminal insertion of stent into renal artery |
| 2 | L471 | OPCS-4 | Percutaneous transluminal angioplasty of visceral branch of abdominal aorta NEC |
| 2 | L518 | OPCS-4 | Other specified other bypass of iliac artery |
| 2 | L671 | OPCS-4 | Biopsy of artery NEC |
| 2 | L721 | OPCS-4 | Arteriography NEC |
| 2 | O023 | OPCS-4 | Percutaneous transluminal balloon assisted coil embolisation of single aneurysm of artery |
| 2 | O121 | OPCS-4 | Superficial temporal artery |
| 2 | U117 | OPCS-4 | Magnetic resonance angiography |
| 2 | U213 | OPCS-4 | Positron emission tomography NEC |
| 2 | U362 | OPCS-4 | Positron emission tomography with computed tomography NEC |
| 2 | X292 | OPCS-4 | Continuous intravenous infusion of therapeutic substance |
| 2 | X353 | OPCS-4 | Intravenous immunotherapy |
| 2 | X823 | OPCS-4 | Pulmonary arterial hypertension drugs Band 3 |
| 2 | X921 | OPCS-4 | Cytokine inhibitor drugs Band 1 |
| 2 | Z362 | OPCS-4 | Subclavian artery |
|  |  |  |  |
| 3 | D508 | ICD-10 | Other iron deficiency anaemias |
| 3 | D509 | ICD-10 | Iron deficiency anaemia, unspecified |
| 3 | D649 | ICD-10 | Anaemia, unspecified |
| 3 | E273 | ICD-10 | Drug-induced adrenocortical insufficiency |
| 3 | E611 | ICD-10 | Iron deficiency |
| 3 | G439 | ICD-10 | Migraine, unspecified |
| 3 | H356 | ICD-10 | Retinal haemorrhage |
| 3 | H534 | ICD-10 | Visual field defects |
| 3 | H538 | ICD-10 | Other visual disturbances |
| 3 | I10X | ICD-10 | Essential (primary) hypertension |
| 3 | I110 | ICD-10 | Hypertensive heart disease with (congestive) heart failure |
| 3 | I159 | ICD-10 | Secondary hypertension, unspecified |
| 3 | I209 | ICD-10 | Angina pectoris, unspecified |
| 3 | I214 | ICD-10 | Acute subendocardial myocardial infarction |
| 3 | I219 | ICD-10 | Acute myocardial infarction, unspecified |
| 3 | I251 | ICD-10 | Atherosclerotic heart disease |
| 3 | I258 | ICD-10 | Other forms of chronic ischaemic heart disease |
| 3 | I259 | ICD-10 | Chronic ischaemic heart disease, unspecified |
| 3 | I420 | ICD-10 | Dilated cardiomyopathy |
| 3 | I672 | ICD-10 | Cerebral atherosclerosis |
| 3 | I677 | ICD-10 | Cerebral arteritis, not elsewhere classified |
| 3 | I678 | ICD-10 | Other specified cerebrovascular diseases |
| 3 | I679 | ICD-10 | Cerebrovascular disease, unspecified |
| 3 | I700 | ICD-10 | Atherosclerosis of aorta |
| 3 | I959 | ICD-10 | Hypotension, unspecified |
| 3 | I99X | ICD-10 | Other and unspecified disorders of circulatory system |
| 3 | J81X | ICD-10 | Pulmonary oedema |
| 3 | J90X | ICD-10 | Pleural effusion, not elsewhere classified |
| 3 | K318 | ICD-10 | Other specified diseases of stomach and duodenum |
| 3 | M139 | ICD-10 | Arthritis, unspecified |
| 3 | M255 | ICD-10 | Pain in joint |
| 3 | M359 | ICD-10 | Systemic involvement of connective tissue, unspecified |
| 3 | M790 | ICD-10 | Rheumatism, unspecified |
| 3 | M791 | ICD-10 | Myalgia |
| 3 | M796 | ICD-10 | Pain in limb |
| 3 | N189 | ICD-10 | Chronic kidney disease, unspecified |
| 3 | N19X | ICD-10 | Unspecified kidney failure |
| 3 | N26X | ICD-10 | Unspecified contracted kidney |
| 3 | N271 | ICD-10 | Small kidney, bilateral |
| 3 | N289 | ICD-10 | Disorder of kidney and ureter, unspecified |
| 3 | O994 | ICD-10 | Diseases of the circulatory system complicating pregnancy, childbirth and the puerperium |
| 3 | R000 | ICD-10 | Tachycardia, unspecified |
| 3 | R060 | ICD-10 | Dyspnoea |
| 3 | R072 | ICD-10 | Precordial pain |
| 3 | R073 | ICD-10 | Other chest pain |
| 3 | R074 | ICD-10 | Chest pain, unspecified |
| 3 | R098 | ICD-10 | Other specified symptoms and signs involving the circulatory and respiratory systems |
| 3 | R101 | ICD-10 | Pain localized to upper abdomen |
| 3 | R103 | ICD-10 | Pain localized to other parts of lower abdomen |
| 3 | R104 | ICD-10 | Other and unspecified abdominal pain |
| 3 | R298 | ICD-10 | Other and unspecified symptoms and signs involving the nervous and musculoskeletal systems |
| 3 | R42X | ICD-10 | Dizziness and giddiness |
| 3 | R509 | ICD-10 | Fever, unspecified |
| 3 | R51X | ICD-10 | Headache |
| 3 | R55X | ICD-10 | Syncope and collapse |
| 3 | R590 | ICD-10 | Localized enlarged lymph nodes |
| 3 | R619 | ICD-10 | Hyperhidrosis, unspecified |
| 3 | R630 | ICD-10 | Anorexia |
| 3 | R933 | ICD-10 | Abnormal findings on diagnostic imaging of other parts of digestive tract |
| 3 | Z500 | ICD-10 | Cardiac rehabilitation |
| 3 | Z871 | ICD-10 | Personal history of diseases of the digestive system |
| 3 | Z904 | ICD-10 | Acquired absence of other parts of digestive tract |
| 3 | K401 | OPCS-4 | Saphenous vein graft replacement of one coronary artery |
| 3 | K451 | OPCS-4 | Double anastomosis of mammary arteries to coronary arteries |
| 3 | K491 | OPCS-4 | Percutaneous transluminal balloon angioplasty of one coronary artery |
| 3 | K611 | OPCS-4 | Implantation of cardiac pacemaker system NEC |
| 3 | K633 | OPCS-4 | Angiocardiography of left side of heart NEC |
| 3 | K634 | OPCS-4 | Coronary arteriography using two catheters |
| 3 | K636 | OPCS-4 | Coronary arteriography NEC |
| 3 | L352 | OPCS-4 | Arteriography of cerebral artery |
| 3 | L763 | OPCS-4 | Endovascular placement of two metallic stents |
| 3 | U081 | OPCS-4 | Computed tomography of abdomen NEC |
| 3 | U102 | OPCS-4 | Cardiac computed tomography angiography |
| 3 | U103 | OPCS-4 | Cardiac magnetic resonance imaging |
| 3 | U111 | OPCS-4 | Ultrasound of carotid artery |
| 3 | U114 | OPCS-4 | Computed tomography scan of cerebral vessels |
| 3 | U118 | OPCS-4 | Other specified diagnostic imaging of vascular system |
| 3 | U201 | OPCS-4 | Transthoracic echocardiography |
| 3 | U202 | OPCS-4 | Transoesophageal echocardiography |
| 3 | U297 | OPCS-4 | Short synacthen test |
| 3 | U354 | OPCS-4 | Computed tomography of pulmonary arteries |
| 3 | Y731 | OPCS-4 | Cardiopulmonary bypass |
| 3 | Y798 | OPCS-4 | Other specified approach to organ through artery |
| 3 | Z342 | OPCS-4 | Aortic arch |
| 3 | Z343 | OPCS-4 | Descending thoracic aorta |
| 3 | Z344 | OPCS-4 | Thoracic aorta NEC |
| 3 | Z347 | OPCS-4 | Abdominal aorta NEC |
| 3 | Z348 | OPCS-4 | Specified aorta NEC |
| 3 | Z349 | OPCS-4 | Aorta NEC |
| 3 | Z357 | OPCS-4 | Artery of circle of Willis |
| 3 | Z361 | OPCS-4 | Carotid artery NEC |
| 3 | Z363 | OPCS-4 | Axillary artery |
| 3 | Z364 | OPCS-4 | Brachial artery |
| 3 | Z365 | OPCS-4 | Vertebral artery |
| 3 | Z368 | OPCS-4 | Specified branch of thoracic aorta NEC |
| 3 | Z373 | OPCS-4 | Superior mesenteric artery |
| 3 | Z401 | OPCS-4 | Pulmonary artery |
| 3 | Z976 | OPCS-4 | Iliac artery NEC |

### **Supplementary Table S3:** Code lists for diagnosis date refinement

|  | Summary | ICD-10 codes | OPCS-4 codes |
| --- | --- | --- | --- |
| List 1 | Specific for TAK and probably is | 8 | 0 |
| List 2 | Likely to be associated with TAK | 43 | 24 |
| List 3 | Could be associated with TAK | 62 | 36 |

### **Supplementary Table S4:** Summary of the extent of diagnosis date refinement for incident cases of Takayasu arteritis in England 2006/07 to 2022/23

| Extent of date refinement (years) | Number* (%) | | |
| --- | --- | --- | --- |
|  | Overall | Pre-COVID19 | Post-COVID19 |
| <1 year | 670 (54%) | 495 (58%) | 175 (45%) |
| 1-2 years | 195 (16%) | 140 (17%) | 50 (13%) |
| 3-5 years | 160 (13%) | 110 (13%) | 50 (13%) |
| 6-10 years | 160 (13%) | 85 (10%) | 75 (19%) |
| >10 years | 50 (4%) | 15 (2%) | 35 (9%) |

^*^ Cases rounded to nearest 5 to comply with disclosure control protocol.

### **Supplementary Figure S1:** Comparison of diagnosis date adjustment strategies

Based on occurrence of prior conditions using (estimated diagnosis date – known diagnosis date) in days. Positive offsets therefore indicate an estimated diagnosis date after the known diagnosis.

The best code list only strategy was to use list 1 alone (2nd graph). However, also adjusting by OP clinic appointment (rheumatological, renal or vascular surgery) (bottom graph) improved further.

# Epidemiology of Takayasu Arteritis

## Additional methods

### Ascertainment of IMD from available data – additional details

As there have been iterations of IMD over time we used the IMD that most closely matched the year of underlying deprivation data and the diagnosis year.

Missing LSOA11, for prevalence calculations and diagnoses made in 2016 onwards, were mapped from LSOA01 where available. Otherwise, we used LSOA11 data across multiple datasets using BHF Data Science Centre’s curated LSOA Multisource table[14]. Missing LSOA11s were imputed based on the closest recorded LSOA to the index or diagnosis date. If there were two identical closest LSOAs, HES APC was prioritised, followed by HES OP, HES AE, and finally General Practice Extraction Service (GPES) Data for Pandemic Planning and Research (GDPPR). Missing LSOA01, for diagnoses in 2006-2015, were imputed using all records in HES APC, HES OP, and HES Accident and Emergency (A&E). Date of death was obtained through linkage to the Civil Registration Deaths dataset.

Where LSOA01 was missing, for diagnoses in 2006-2015, we used the closest recorded LSOA01 to the diagnosis date from HES APC, HES OP, or HES AE.

## Additional results

### **Supplementary Table S5:** Overall incidence of Takayasu arteritis (per million people) by ethnicity: sensitivity analysis assuming all individuals with unknown ethnicity are White

|  |  | Cases^1^ | Crude incidence rate ratios  (95% CI)^2^ | Adjusted incidence rate ratios  (95% CI)^3^ |
| --- | --- | --- | --- | --- |
| **Ethnicity** | |  |  |  |
|  | Asian | 140 (11%) | 1.4 (1.1, 1.6) | 1.7 (1.4, 2) |
|  | Black | 40 (3%) | 0.9 (0.6, 1.2) | 1.1 (0.8, 1.5) |
|  | Mixed | 10 (1%) | 0.3 (0.2, 0.6) | 0.9 (0.5, 1.4) |
|  | White | 1005 (81%) | 1 | 1 |
|  | Other | 40 (3%) | 2.2 (1.6, 3) | 3 (2.2, 4) |

^1^ Cases rounded to nearest 5 to comply with disclosure control protocol

^2^ Confidence intervals calculated using the standard error of the log incidence rate ratio

^3^ Incidence rate ratios adjusted for age and gender

### **Supplementary Table S6:** Overall incidence of Takayasu arteritis (per million people): post-hoc analysis where age and ethnicity are modelled as an interaction using Poisson regression

|  |  | Number of cases^1^ | | Adjusted incidence rate ratios (95% CI)^2,3^ | |
| --- | --- | --- | --- | --- | --- |
|  |  | Individuals **not** of White ethnicity | Individuals of White ethnicity | Individuals **not** of White ethnicity | Individuals of White ethnicity |
| **Age group (years)** | |  |  |  |  |
|  | 0-15 | 30 | 35 | 0.3 (0.2, 0.5) | 0.4 (0.2, 0.5) |
|  | 16-39 | 140 | 190 | 1 | 1 |
|  | 40-54 | 70 | 185 | 0.9 (0.6, 1.3) | 1.4 (1.1, 1.7) |
|  | 55-69 | 60 | 280 | 1.3 (0.8, 1.9) | 2.4 (2, 2.9) |
|  | 70-84 | 20 | 190 | 1.6 (0.9, 2.6) | 2.5 (2.1, 3.1) |
|  | 85+ | suppressed | 30 | 5.4 (2.4, 10.4) | 1.6 (1.1, 2.3) |

^1^ Cases rounded to nearest 5 (or suppressed if less than 10) to comply with disclosure control protocol

^2^ Confidence intervals calculated using the standard error of the log incidence rate ratio

^3^ Incidence rate ratios adjusted for sex and age group * binary ethnicity. A likelihood ratio test comparing this to a non-interaction model with sex, age, and binary ethnicity, was statistically significant (p=0.0005).

### **Supplementary Table S7:** Overall incidence of Takayasu arteritis (per million people): sensitivity analysis where age and ethnicity are modelled as an interaction using the negative binomial distribution

|  |  | Adjusted incidence rate ratios (95% CI)^1,2^ | |
| --- | --- | --- | --- |
|  |  | Individuals **not** of White ethnicity | Individuals of White ethnicity |
| **Age group (years)** | |  |  |
|  | 0-15 | 0.4 (0.2, 0.7) | 0.4 (0.2, 0.8) |
|  | 16-39 | 1 | 1 |
|  | 40-54 | 1.1 (0.7, 1.8) | 1.4 (0.8, 2.6) |
|  | 55-69 | 1.5 (0.9, 2.5) | 2.5 (1.4, 4.6) |
|  | 70-84 | 2 (1, 3.7) | 2.8 (1.5, 5.1) |
|  | 85+ | 6.8 (2.8, 14.3) | 2.2 (1.1, 4.5) |

^1^ Confidence intervals calculated using the standard error of the log incidence rate ratio

^2^ Incidence rate ratios adjusted for sex and age group * binary ethnicity

### **Supplementary Table S8:** Overall incidence of Takayasu arteritis (per million people): sensitivity analysis of age- and sex-specific incidence rate ratios derived from multivariable models with ethnicity, IMD, and region

|  |  | Adjusted incidence rate ratios  (95% CI)^1^ | | | | | | |  |
| --- | --- | --- | --- | --- | --- | --- | --- | --- | --- |
|  |  | Adjusted by ethnicity (nominal) | | Adjusted by ethnicity (binary) | | Adjusted by IMD | | Adjusted by region | |
| **Gender** | |  | |  | |  | |  | |
|  | Female | 2.6 (2.3, 3) | | 2.6 (2.3, 3) | | 2.7 (2.4, 3) | | 2.6 (2.3, 3) | |
|  | Male | 1 | | 1 | | 1 | | 1 | |
| **Age group (years)** | | |  | |  | |  |  |  |
|  | 0-15 | 0.4 (0.3, 0.5) | | 0.4 (0.3, 0.5) | | 0.3 (0.3, 0.4) | | 0.4 (0.3, 0.5) | |
|  | 16-39 | 1 | | 1 | | 1 | | 1 | |
|  | 40-54 | 1.2 (1, 1.5) | | 1.2 (1, 1.5) | | 1.2 (1, 1.4) | | 1.2 (1, 1.4) | |
|  | 55-69 | 2.1 (1.8, 2.5) | | 2.1 (1.8, 2.5) | | 2 (1.7, 2.3) | | 2 (1.7, 2.3) | |
|  | 70-84 | 2.3 (1.9, 2.7) | | 2.3 (1.9, 2.7) | | 2 (1.7, 2.3) | | 2 (1.7, 2.4) | |
|  | 85+ | 1.8 (1.2, 2.4) | | 1.8 (1.2, 2.4) | | 1.3 (0.9, 1.8) | | 1.4 (1, 1.9) | |

^1^ Confidence intervals calculated using the standard error of the log prevalence rate ratio. In addition to the variables indicated in the column headers, age-specific rates are adjusted by sex and sex-specific rates are adjusted by age.

### **Supplementary Table S9:** Overall prevalence of Takayasu arteritis (per million people) by ethnicity: sensitivity analysis assuming all individuals with unknown ethnicity are White

|  |  | Cases^1^ | Crude prevalence rate ratios  (95% CI)^2^ | Adjusted prevalence rate ratios  (95% CI)^3^ |
| --- | --- | --- | --- | --- |
| **Ethnicity** | |  |  |  |
|  | Asian | 165 (13%) | 1.4 (1.1, 1.6) | 1.9 (1.6, 2.2) |
|  | Black | 50 (4%) | 0.9 (0.7, 1.2) | 1.3 (0.9, 1.7) |
|  | Mixed | 15 (1%) | 0.4 (0.2, 0.7) | 0.9 (0.6, 1.5) |
|  | White | 1020 (79%) | 1 | 1 |
|  | Other | 40 (3%) | 1.5 (1.1, 2) | 2.2 (1.6, 2.9) |

^1^ Cases rounded to nearest 5 to comply with disclosure control protocol

^2^ Confidence intervals calculated using the standard error of the log prevalence rate ratio

^3^ Prevalence rate ratios adjusted for age and gender

### **Supplementary Table S10:** Overall prevalence of Takayasu arteritis (per million people): post-hoc analysis where age and ethnicity are modelled as an interaction using Poisson regression

|  |  | Number of cases^1^ | | Adjusted prevalence rate ratios (95% CI)^2,3^ | |
| --- | --- | --- | --- | --- | --- |
|  |  | Individuals **not** of White ethnicity | Individuals of White ethnicity | Individuals **not** of White ethnicity | Individuals of White ethnicity |
| **Age group (years)** | |  |  |  |  |
|  | 0-15 | 15 | 15 | 0.3 (0.2, 0.5) | 0.2 (0.1, 0.3) |
|  | 16-39 | 95 | 140 | 1 | 1 |
|  | 40-54 | 115 | 175 | 2.3 (1.7, 3.1) | 1.9 (1.5, 2.3) |
|  | 55-69 | 80 | 270 | 3 (2.1, 4.1) | 2.9 (2.4, 3.6) |
|  | 70-84 | 50 | 280 | 3.9 (2.4, 5.9) | 4.4 (3.6, 5.4) |
|  | 85+ | suppressed | 50 | 6 (2.7, 11.7) | 3.3 (2.4, 4.5) |

^1^ Cases rounded to nearest 5 (or suppressed if less than 10) to comply with disclosure control protocol

^2^ Confidence intervals calculated using the standard error of the log prevalence rate ratio

^3^ Prevalence rate ratios adjusted for sex and age group * binary ethnicity. A likelihood ratio test comparing this to a non-interaction model with sex, age, and binary ethnicity, was not significant (p=0.3).

There was minimal overdispersion, so this was not repeated using the negative binomial.

### **Supplementary Table S11:** Sensitivity analysis of crude incidence and prevalence rates to account for the uncertainty introduced by rounding of case counts.

| **Table** | **Variable** | **Year** | **Population** | **Rounded count** | **Rounded estimate** | **Lowest possible count** | **Lowest estimate** | **Highest possible count** | **Highest estimate** |
| --- | --- | --- | --- | --- | --- | --- | --- | --- | --- |
| 2 | Year | 2006/07 | 50,965,186 | 70 | 1.4 (1.1, 1.7) | 68 | 1.3 (1, 1.7) | 72 | 1.4 (1.1, 1.8) |
| 2 | Year | 2007/08 | 51,381,093 | 80 | 1.6 (1.2, 1.9) | 78 | 1.5 (1.2, 1.9) | 82 | 1.6 (1.3, 2) |
| 2 | Year | 2008/09 | 51,815,853 | 65 | 1.3 (1, 1.6) | 63 | 1.2 (0.9, 1.6) | 67 | 1.3 (1, 1.6) |
| 2 | Year | 2009/10 | 52,196,381 | 75 | 1.4 (1.1, 1.8) | 73 | 1.4 (1.1, 1.8) | 77 | 1.5 (1.2, 1.8) |
| 2 | Year | 2010/11 | 52,642,452 | 60 | 1.1 (0.9, 1.5) | 58 | 1.1 (0.8, 1.4) | 62 | 1.2 (0.9, 1.5) |
| 2 | Year | 2011/12 | 53,107,169 | 70 | 1.3 (1, 1.7) | 68 | 1.3 (1, 1.6) | 72 | 1.4 (1.1, 1.7) |
| 2 | Year | 2012/13 | 53,506,812 | 85 | 1.6 (1.3, 2) | 83 | 1.6 (1.2, 1.9) | 87 | 1.6 (1.3, 2) |
| 2 | Year | 2013/14 | 53,918,686 | 90 | 1.7 (1.3, 2.1) | 88 | 1.6 (1.3, 2) | 92 | 1.7 (1.4, 2.1) |
| 2 | Year | 2014/15 | 54,370,319 | 70 | 1.3 (1, 1.6) | 68 | 1.3 (1, 1.6) | 72 | 1.3 (1, 1.7) |
| 2 | Year | 2015/16 | 54,808,676 | 80 | 1.5 (1.2, 1.8) | 78 | 1.4 (1.1, 1.8) | 82 | 1.5 (1.2, 1.9) |
| 2 | Year | 2016/17 | 55,289,034 | 65 | 1.2 (0.9, 1.5) | 63 | 1.1 (0.9, 1.5) | 67 | 1.2 (0.9, 1.5) |
| 2 | Year | 2017/18 | 55,619,548 | 75 | 1.3 (1.1, 1.7) | 73 | 1.3 (1, 1.7) | 77 | 1.4 (1.1, 1.7) |
| 2 | Year | 2018/19 | 55,924,528 | 80 | 1.4 (1.1, 1.8) | 78 | 1.4 (1.1, 1.7) | 82 | 1.5 (1.2, 1.8) |
| 2 | Year | 2019/20 | 56,230,056 | 75 | 1.3 (1, 1.7) | 73 | 1.3 (1, 1.6) | 77 | 1.4 (1.1, 1.7) |
| 2 | Year | 2020/21 | 56,325,961 | 55 | 1 (0.7, 1.3) | 53 | 0.9 (0.7, 1.2) | 57 | 1 (0.8, 1.3) |
| 2 | Year | 2021/22 | 56,554,891 | 65 | 1.1 (0.9, 1.5) | 63 | 1.1 (0.9, 1.4) | 67 | 1.2 (0.9, 1.5) |
| 2 | Year | 2022/23 | 57,106,398 | 75 | 1.3 (1, 1.6) | 73 | 1.3 (1, 1.6) | 77 | 1.3 (1.1, 1.7) |
| 2 | Year | Total | 921,763,043 | 1235 | 1.3 (1.3, 1.4) | 1233 | 1.3 (1.3, 1.4) | 1237 | 1.3 (1.3, 1.4) |
| 3 | Overall | Total | 921,763,043 | 1235 | 1.3 (1.3, 1.4) | 1233 | 1.3 (1.3, 1.4) | 1237 | 1.3 (1.3, 1.4) |
| 3 | Gender | Female | 469,260,686 | 910 | 1.9 (1.8, 2.1) | 908 | 1.9 (1.8, 2.1) | 912 | 1.9 (1.8, 2.1) |
| 3 | Gender | Male | 452,502,357 | 325 | 0.7 (0.6, 0.8) | 323 | 0.7 (0.6, 0.8) | 327 | 0.7 (0.6, 0.8) |
| 3 | Age group (years) | 0-15 | 173,860,290 | 65 | 0.4 (0.3, 0.5) | 63 | 0.4 (0.3, 0.5) | 67 | 0.4 (0.3, 0.5) |
| 3 | Age group (years) | 16-39 | 291,516,886 | 325 | 1.1 (1, 1.2) | 323 | 1.1 (1, 1.2) | 327 | 1.1 (1, 1.3) |
| 3 | Age group (years) | 40-54 | 188,919,934 | 255 | 1.3 (1.2, 1.5) | 253 | 1.3 (1.2, 1.5) | 257 | 1.4 (1.2, 1.5) |
| 3 | Age group (years) | 55-69 | 153,900,256 | 340 | 2.2 (2, 2.5) | 338 | 2.2 (2, 2.4) | 342 | 2.2 (2, 2.5) |
| 3 | Age group (years) | 70-84 | 92,290,470 | 210 | 2.3 (2, 2.6) | 208 | 2.3 (2, 2.6) | 212 | 2.3 (2, 2.6) |
| 3 | Age group (years) | 85+ | 21,275,207 | 35 | 1.6 (1.1, 2.3) | 33 | 1.6 (1.1, 2.2) | 37 | 1.7 (1.2, 2.4) |
| 3 | Ethnicity | Asian | 79,079,641 | 140 | 1.8 (1.5, 2.1) | 138 | 1.7 (1.5, 2.1) | 142 | 1.8 (1.5, 2.1) |
| 3 | Ethnicity | Black | 34,988,125 | 40 | 1.1 (0.8, 1.6) | 38 | 1.1 (0.8, 1.5) | 42 | 1.2 (0.9, 1.6) |
| 3 | Ethnicity | Mixed | 23,510,792 | 10 | 0.4 (0.2, 0.8) | 8 | 0.3 (0.1, 0.7) | 12 | 0.5 (0.3, 0.9) |
| 3 | Ethnicity | White | 770,162,737 | 910 | 1.2 (1.1, 1.3) | 908 | 1.2 (1.1, 1.3) | 912 | 1.2 (1.1, 1.3) |
| 3 | Ethnicity | Other | 14,021,748 | 40 | 2.9 (2, 3.9) | 38 | 2.7 (1.9, 3.7) | 42 | 3 (2.2, 4) |
| 3 | IMD | 1 | 184,652,849 | 265 | 1.4 (1.3, 1.6) | 263 | 1.4 (1.3, 1.6) | 267 | 1.4 (1.3, 1.6) |
| 3 | IMD | 2 | 187,920,743 | 240 | 1.3 (1.1, 1.4) | 238 | 1.3 (1.1, 1.4) | 242 | 1.3 (1.1, 1.5) |
| 3 | IMD | 3 | 185,846,260 | 245 | 1.3 (1.2, 1.5) | 243 | 1.3 (1.1, 1.5) | 247 | 1.3 (1.2, 1.5) |
| 3 | IMD | 4 | 182,894,529 | 215 | 1.2 (1, 1.3) | 213 | 1.2 (1, 1.3) | 217 | 1.2 (1, 1.4) |
| 3 | IMD | 5 | 180,434,196 | 270 | 1.5 (1.3, 1.7) | 268 | 1.5 (1.3, 1.7) | 272 | 1.5 (1.3, 1.7) |
| 3 | Region | North East | 44,343,300 | 60 | 1.4 (1, 1.7) | 58 | 1.3 (1, 1.7) | 62 | 1.4 (1.1, 1.8) |
| 3 | Region | North West | 121,869,783 | 160 | 1.3 (1.1, 1.5) | 158 | 1.3 (1.1, 1.5) | 162 | 1.3 (1.1, 1.6) |
| 3 | Region | Yorkshire and The Humber | 90,882,121 | 130 | 1.4 (1.2, 1.7) | 128 | 1.4 (1.2, 1.7) | 132 | 1.5 (1.2, 1.7) |
| 3 | Region | East Midlands | 79,069,366 | 90 | 1.1 (0.9, 1.4) | 88 | 1.1 (0.9, 1.4) | 92 | 1.2 (0.9, 1.4) |
| 3 | Region | West Midlands | 97,244,072 | 90 | 0.9 (0.7, 1.1) | 88 | 0.9 (0.7, 1.1) | 92 | 0.9 (0.8, 1.2) |
| 3 | Region | East of England | 102,334,136 | 175 | 1.7 (1.5, 2) | 173 | 1.7 (1.4, 2) | 177 | 1.7 (1.5, 2) |
| 3 | Region | London | 143,059,361 | 210 | 1.5 (1.3, 1.7) | 208 | 1.5 (1.3, 1.7) | 212 | 1.5 (1.3, 1.7) |
| 3 | Region | South East | 150,577,955 | 190 | 1.3 (1.1, 1.5) | 188 | 1.2 (1.1, 1.4) | 192 | 1.3 (1.1, 1.5) |
| 3 | Region | South West | 92,382,949 | 130 | 1.4 (1.2, 1.7) | 128 | 1.4 (1.2, 1.6) | 132 | 1.4 (1.2, 1.7) |
| 4 | Overall | Total | 57,106,398 | 1285 | 22.5 (21.3, 23.8) | 1283 | 22.5 (21.3, 23.7) | 1287 | 22.5 (21.3, 23.8) |
| 4 | Gender | Female | 29,123,108 | 1000 | 34.3 (32.2, 36.5) | 998 | 34.3 (32.2, 36.5) | 1002 | 34.4 (32.3, 36.6) |
| 4 | Gender | Male | 27,983,290 | 285 | 10.2 (9, 11.4) | 283 | 10.1 (9, 11.4) | 287 | 10.3 (9.1, 11.5) |
| 4 | Age group (years) | 0-15 | 10,575,320 | 30 | 2.8 (1.9, 4) | 28 | 2.6 (1.8, 3.8) | 32 | 3 (2.1, 4.3) |
| 4 | Age group (years) | 16-39 | 17,634,431 | 235 | 13.3 (11.7, 15.1) | 233 | 13.2 (11.6, 15) | 237 | 13.4 (11.8, 15.3) |
| 4 | Age group (years) | 40-54 | 11,031,939 | 290 | 26.3 (23.3, 29.5) | 288 | 26.1 (23.2, 29.3) | 292 | 26.5 (23.5, 29.7) |
| 4 | Age group (years) | 55-69 | 10,060,648 | 350 | 34.8 (31.2, 38.6) | 348 | 34.6 (31.1, 38.4) | 352 | 35 (31.4, 38.8) |
| 4 | Age group (years) | 70-84 | 6,379,189 | 325 | 50.9 (45.6, 56.8) | 323 | 50.6 (45.3, 56.5) | 327 | 51.3 (45.9, 57.1) |
| 4 | Age group (years) | 85+ | 1,424,871 | 55 | 38.6 (29.1, 50.2) | 53 | 37.2 (27.9, 48.7) | 57 | 40 (30.3, 51.8) |
| 4 | Ethnicity | Asian | 5,426,315 | 165 | 30.4 (25.9, 35.4) | 163 | 30 (25.6, 35) | 167 | 30.8 (26.3, 35.8) |
| 4 | Ethnicity | Black | 2,381,620 | 50 | 21 (15.6, 27.7) | 48 | 20.2 (14.9, 26.7) | 52 | 21.8 (16.3, 28.6) |
| 4 | Ethnicity | Mixed | 1,669,140 | 15 | 9 (5, 14.8) | 13 | 7.8 (4.1, 13.3) | 17 | 10.2 (5.9, 16.3) |
| 4 | Ethnicity | White | 45,783,290 | 930 | 20.3 (19, 21.7) | 928 | 20.3 (19, 21.6) | 932 | 20.4 (19.1, 21.7) |
| 4 | Ethnicity | Other | 1,229,125 | 40 | 32.5 (23.2, 44.3) | 38 | 30.9 (21.9, 42.4) | 42 | 34.2 (24.6, 46.2) |
| 4 | IMD | 1 | 11,295,065 | 260 | 23 (20.3, 26) | 258 | 22.8 (20.1, 25.8) | 262 | 23.2 (20.5, 26.2) |
| 4 | IMD | 2 | 11,626,962 | 235 | 20.2 (17.7, 23) | 233 | 20 (17.5, 22.8) | 237 | 20.4 (17.9, 23.2) |
| 4 | IMD | 3 | 11,533,486 | 260 | 22.5 (19.9, 25.5) | 258 | 22.4 (19.7, 25.3) | 262 | 22.7 (20, 25.6) |
| 4 | IMD | 4 | 11,254,837 | 240 | 21.3 (18.7, 24.2) | 238 | 21.1 (18.5, 24) | 242 | 21.5 (18.9, 24.4) |
| 4 | IMD | 5 | 11,027,739 | 290 | 26.3 (23.4, 29.5) | 288 | 26.1 (23.2, 29.3) | 292 | 26.5 (23.5, 29.7) |
| 4 | Region | North East | 2,683,040 | 60 | 22.4 (17.1, 28.8) | 58 | 21.6 (16.4, 27.9) | 62 | 23.1 (17.7, 29.6) |
| 4 | Region | North West | 7,516,113 | 165 | 22 (18.7, 25.6) | 163 | 21.7 (18.5, 25.3) | 167 | 22.2 (19, 25.9) |
| 4 | Region | Yorkshire and The Humber | 5,541,262 | 135 | 24.4 (20.4, 28.8) | 133 | 24 (20.1, 28.4) | 137 | 24.7 (20.8, 29.2) |
| 4 | Region | East Midlands | 4,934,939 | 95 | 19.3 (15.6, 23.5) | 93 | 18.8 (15.2, 23.1) | 97 | 19.7 (15.9, 24) |
| 4 | Region | West Midlands | 6,021,653 | 100 | 16.6 (13.5, 20.2) | 98 | 16.3 (13.2, 19.8) | 102 | 16.9 (13.8, 20.6) |
| 4 | Region | East of England | 6,398,497 | 170 | 26.6 (22.7, 30.9) | 168 | 26.3 (22.4, 30.5) | 172 | 26.9 (23, 31.2) |
| 4 | Region | London | 8,866,180 | 235 | 26.5 (23.2, 30.1) | 233 | 26.3 (23, 29.9) | 237 | 26.7 (23.4, 30.4) |
| 4 | Region | South East | 9,379,833 | 200 | 21.3 (18.5, 24.5) | 198 | 21.1 (18.3, 24.3) | 202 | 21.5 (18.7, 24.7) |
| 4 | Region | South West | 5,764,881 | 125 | 21.7 (18, 25.8) | 123 | 21.3 (17.7, 25.5) | 127 | 22 (18.4, 26.2) |

### **Supplementary Table S12:** Summary of NHS providers’ TAK coding habits in the incident cohort.

|  | **Overall** | **Pre-COVID-19*** | **Post-COVID-19*** |
| --- | --- | --- | --- |
| Unique providers who have coded TAK (PROCODE3) | 173 (100%) | 168 (100%) | 111 (100%) |
| Providers coding in HES OP | 23 (13%) | 19 (11%) | 11 (10%) |

*Based on whether first TAK code appeared before or after 11th March 2020.

### **Supplementary Figure S2:** Flow diagram of cohort construction from HES Admitted Patient Care (APC) and HES Outpatients (OP) data

Unique patients in HES APC

(n = 74,875,648)

Unique patients in HES OP

(n = 79,886,059)

Patients with ICD-10 code M31.4

(n = 2,005)

Patients with ICD-10 code M31.4

(n = 326)

Patients aged ≥ 2 years at diagnosis

(n = 1,792)

Total patients with ICD-10 code M31.4

(n = 1,845)

Patients aged < 2 years at diagnosis

(n = 53)

Incident cases in England 2006-2023

(n = 1,235)

Patients diagnosed before 1^st^ April 2006 or after 31^st^ March 2023, or not in England at diagnosis

(n = 557)

Prevalent cases in England Mid-2022

(n = 1,285)

Patients diagnosed after, deceased before, or not resident in England on 1^st^ July 2022

(n = 507)

Patients with diagnosis date refinement ≥ year*

(n = 565)

Patients with diagnosis date refinement ≥ year*

(n = 670)

Patients with valid NHS ID

(n = 301)

Patients with valid NHS ID

(n = 1,721)

Patients not already in HES APC

(n = 124)

***** Cases rounded to nearest 5 to comply with disclosure control protocol

### **Supplementary Figure S3:** Median age in years of incident cases of Takayasu arteritis (IQR)

by year of diagnosis

### **Supplementary Figure S4:** Region of residence of prevalent cases of Takayasu arteritis: white versus other/unknown ethnicity

### **Supplementary Figure S5:** Ethnicity of prevalent cases of Takayasu arteritis: London residents versus other

### **Supplementary Figure S6:** IMD of prevalent cases of Takayasu arteritis by region (IMD 1 = most deprived; 5 = least)

### **Supplementary Figure S7:** IMD of prevalent cases of Takayasu arteritis by ethnicity (IMD 1 = most deprived; 5 = least)
