# Supplementary material for: Incidence and prevalence of Takayasu arteritis in England before and after the COVID-19 pandemic: results from RECORDER
Source: Rheumatol Adv Pract. 2026 Jul 24;10(3):rkag078. doi: 10.1093/rap/rkag078 (PMC13493044; doi:10.1093/rap/rkag078)
Supplement: Supplementary file 1 [file rkag078_supplementary_data.zip › CVD-COVID-UK & COVID-IMPACT Consortium Members.pdf]

CVD-COVID-UK / COVID-IMPACT Consortium Members (03/04/2026)

British Heart Foundation Data Science Centre
Led by Health Data Research UK

Institution	Member Name
Barts Health NHS Trust	Alastair Proudfoot
Barts Health NHS Trust	Andrew Constantine
Barts Health NHS Trust	Dan Jones
Barts Health NHS Trust	Krishnaraj Rathod
Barts Health NHS Trust	Nida Ahmed
Barts Health NHS Trust	Richard Fitzgerald
British Heart Foundation	Dan O’Connell
British Heart Foundation	Rony Arafin
British Heart Foundation	Sonya Babu-Narayan
Cancer Research UK	Jon Shelton
Cancer Research UK	Martina Slapkova
Cancer Research UK	Rosie Hinchliffe
Cancer Research UK	Shane Johnson
Cardiff Metropolitan University	Renin Toms
Cardiff University	Julia Townson
European Bioinformatics Institute	Ewan Birney
European Bioinformatics Institute	Moritz Gerstung
European Bioinformatics Institute	Tomas Fitzgerald
Great Ormond Street Hospital	Katherine Brown
Guy’s and St Thomas’ NHS Foundation Trust	Benjamin Zuckerman
Guy’s and St Thomas’ NHS Foundation Trust	Ernest Wong
Guy’s and St Thomas’ NHS Foundation Trust	Lily Benton
Guy's and St Thomas' NHS Foundation Trust	Tasanee Braithwaite
Health Data Research UK	Alexis Webb
Health Data Research UK/BHF Data Science Centre	Anna Stevenson
Health Data Research UK/BHF Data Science Centre	Cathie Sudlow
Health Data Research UK/BHF Data Science Centre	Fionna Chalmers
Health Data Research UK/BHF Data Science Centre	Jadene Lewis
Health Data Research UK/BHF Data Science Centre	John Nolan
Health Data Research UK/BHF Data Science Centre	Lars Murdock
Health Data Research UK/BHF Data Science Centre	Laura Sherlock

Institution	Member Name
Health Data Research UK/BHF Data Science Centre	Lynn Morrice
Health Data Research UK/BHF Data Science Centre	Mehrdad Mizani
Health Data Research UK/BHF Data Science Centre	Melissa Webb
Health Data Research UK/BHF Data Science Centre	Ross Forsyth
Health Data Research UK/BHF Data Science Centre	Samaira Khan
Health Data Research UK/BHF Data Science Centre	Sandy Wibrewdc
Health Data Research UK/BHF Data Science Centre	Steffen Petersen
Health Data Research UK/BHF Data Science Centre	Thomas Bolton
Health Data Research UK/BHF Data Science Centre	Zach Welshman
Healthcare Quality Improvement Partnership	Caroline Rogers
Heidelberg University	Petr Andriushchenko
Hull York Medical School	Donna Wakefield
Imperial College London	Ahmed Salih
Imperial College London	Alistair Marsland
Imperial College London	Alun Davies
Imperial College London	Arunashis Sau
Imperial College London	Costas Kallis
Imperial College London	Francesca Cavallo
Imperial College London	Fu Siong Ng
Imperial College London	Hannah Whittaker
Imperial College London	Ioanna Tzoulaki
Imperial College London	Jennifer Quint
Imperial College London	Juliette Unwin
Imperial College London	Libor Pastika
Imperial College London	Petter Brodin
Imperial College London	Philip Stone
Imperial College London	Safa Salim
Imperial College London	Sarah Cook
Imperial College London	Sarah Onida
Imperial College London (LCP)	Alistair Marsland

Institution	Member Name
Imperial College London (LCP)	Andrew Thompson
Imperial College London (LCP)	Sara Holloway
Imperial College London (LCP)	Thomas Porter
Keele University	Mamas Mamas
King's College London	Abdel Douiri
King's College London	Adejoke Oluyase
King's College London	Ajay Shah
King's College London	Alexandru Dregan
King’s College London	Amy Ronaldson
King's College London	Anna Bone
King's College London	Antonio Cannata
King's College London	Ben Bray
King's College London	Charles Wolfe
King's College London	Daniel Bromage
King's College London	Dominic Oliver
King's College London	Elena Nikiphorou
King's College London	Emeka Chukwusa
King’s College London	Emel Yorganci
King's College London	Gareth Williams
King's College London	Gayan Perera
King's College London	Harry Watson
King’s College London	Iain Marshall
King’s College London	India Tunnard
King's College London	Irene Higginson
King's College London	Javiera Leniz Martelli
King's College London	Jayati Das-Munshi
King's College London	Joanna Davies
King's College London	Johnny Downs
King’s College London	Jonathan Hudson
King's College London	Katherine Sleeman
King’s College London	Laia Becares
King’s College London	Linglong Qian
King’s College London	Matthew O'Connell

Institutions party to the Data Sharing Agreement with NHS England for access to the data in the Secure Data Environment service for England for CVD-COVID-UK/COVID-IMPACT are: Imperial College London, King's College London, London School of Hygiene & Tropical Medicine, Swansea University, University College London, University of Bristol, University of Cambridge, University of Dundee, University of Glasgow, University of Leicester, University of Liverpool, University of Manchester, University of Nottingham, University of Oxford, University of Sheffield, University of Southampton.

CVD-COVID-UK / COVID-IMPACT Consortium Members (03/04/2026)

British Heart Foundation Data Science Centre
Led by Health Data Research UK

Institution	Member Name
King's College London	Mevhibe Hocaoglu
King's College London	Natasha Chilman
King's College London	Rachel Cripps
King's College London	Richard Killick
King's College London	Theresa McDonagh
King's College London	Vasa Curcin
King's College London	Zeljko Kraljevic
London School of Economics & Political Science	Rocco Friebel
London School of Hygiene & Tropical Medicine	Akshay Jagadeesh
London School of Hygiene & Tropical Medicine	Antonio Gasparrini
London School of Hygiene & Tropical Medicine	Arturo de la Cruz
London School of Hygiene & Tropical Medicine	Dorothea Nitsch
London School of Hygiene & Tropical Medicine	Julian Matthewman
London School of Hygiene & Tropical Medicine	Neil Pearce
London School of Hygiene & Tropical Medicine	Patrick Bidulka
London School of Hygiene & Tropical Medicine	Qiuju Li
London School of Hygiene & Tropical Medicine	Sinéad Langan
London School of Hygiene & Tropical Medicine	Thiago Silva
London School of Hygiene & Tropical Medicine	Zhengnan Zhu
Manchester University NHS Foundation Trust	Martin Rutter
Newcastle-upon-Tyne Hospitals NHS Foundation Trust	Alex Grundmann
NHS England	Adam Hollings
NHS England	Alistair Jones
NHS England	Angeliki Antonarou
NHS England	Daniel Schofield
NHS England	Deborah Lowe
NHS England	Elizabeth Kelly
NHS England	Richardson
NHS England	Humaira Hussein
NHS England	Nickie Wareing
NHS England	Shoaib Ali Ajaib
NHS Lanarkshire	Mark Barber

Institution	Member Name
NHS Scotland	Carole Morris
NICE	Felix Greaves
NICE	Jennifer Beveridge
NICE	Shaun Rowark
NICE	Thomas Lawrence
NICE	Vandana Ayyar-Gupta
Office for National Statistics	Vahé Nafilyan
Queen Mary University of London	Deepti Gurdasani
Queen's University Belfast	Frank Kee
Royal Brompton and Harefield Hospitals	Paz Tayal
Royal College of Surgeons of England	David Cromwell
Royal Free London NHS Foundation Trust	Amar Shah
Royal Free London NHS Foundation Trust	Swapna Mandal
Royal Papworth Hospital NHS Foundation Trust	Florian Falter
Royal Papworth Hospital NHS Foundation Trust	Joseph Newman
Royal United Hospitals Bath NHS Foundation Trust	Jennifer Rossdale
St George's University of London	Baboucarr Njie
St George's University of London	Elijah Behr
St George's University of London	Nuria Sanchez
St George's University of London	Rosie Freer
St George's University of London	Xinkai Wang
Swansea Bay University Health Board	Daniel Harris
Swansea University	Amanda Marchant
Swansea University	Ashley Akbari
Swansea University	Daniel King
Swansea University	David Powell
Swansea University	Elizabeth A Ellins
Swansea University	Fatemeh Torabi
Swansea University	Gareth Davies
Swansea University	Hoda Abbasizanjani
Swansea University	Huw Strafford
Swansea University	Jane Lyons
Swansea University	Julian Halcox

Institution	Member Name
Swansea University	Laura North
Swansea University	Marcos del Pozo Banos
Swansea University	Owen Pickrell
Swansea University	Ronan Lyons
Swansea University - DATAMIND	Ann John
University College	Robert Aldridge
University College London	Abraham Olvera-Barrios
University College London	Adnan Tufail
University College London	Alasdair Warwick
University College London	Alex Handy
University College London	Alexei Yavlinsky
University College London	Alvina Lai
University College London	Ami Banerjee
University College London	Ana Torralbo
University College London	Ana-Catarina Pinho-Gomes
University College London	Andrej Ivanovic
University College London	Andrew Lambarth
University College London	Anthony Khawaja
University College London	Ashkan Dashtban
University College London	Ashley Dickson
University College London	Becky White
University College London	Christina Pagel
University College London	Christopher Tomlinson
University College London	Chu Siyu
University College London	David Selby
University College London	Emma Whitfield
University College London	Eva Keller
University College London	Evaleen Malgapo
University College London	Ferran Espuny-Pujol
University College London	Flavien Hardy
University College London	Floriaan Schmidt
University College London	Freya Allery
University College London	Harry Hemingway

Institutions party to the Data Sharing Agreement with NHS England for access to the data in the Secure Data Environment service for England for CVD-COVID-UK/COVID-IMPACT are: Imperial College London, King's College London, London School of Hygiene & Tropical Medicine, Swansea University, University College London, University of Bristol, University of Cambridge, University of Dundee, University of Glasgow, University of Leicester, University of Liverpool, University of Manchester, University of Nottingham, University of Oxford, University of Sheffield, University of Southampton.

CVD-COVID-UK / COVID-IMPACT Consortium Members (03/04/2026)

British Heart Foundation Data Science Centre
Led by Health Data Research UK

Institution	Member Name
**University College London**	Helen Richardson
**University College London**	Hiroyuki Yoshimura
**University College London**	Honghan Wu
**University College London**	Jinge Wu
**University College London**	Johan Thygesen
**University College London**	Johannes Heyl
**University College London**	Julia Ive
**University College London**	Kate Cheema
**University College London**	Katie Harron
**University College London**	Ken Li
**University College London**	Kerrie Stevenson
**University College London**	Laura Pasea
**University College London**	Louise Choo
**University College London**	Luca Grieco
**University College London**	Manuel Gomes
**University College London**	Mehrdad Mizani
**University College London**	Mengyun Liu
**University College London**	Michalis Katsoulis
**University College London**	Mohamed Mohamed
**University College London**	Naomi Launders
**University College London**	Nushrat Khan
**University College London**	Paula Lorgelly
**University College London**	Pedro Machado
**University College London**	Pia Hardelid
**University College London**	Qi Huang
**University College London**	Riyaz Patel
**University College London**	Roy Schwartz
**University College London**	Rui Providencia
**University College London**	Ruth Gilbert
**University College London**	Samuel Kim
**University College London**	Scott Isherwood
**University College London**	Simon Ellershaw
**University College London**	Sonya Crowe

Institution	Member Name
**University College London**	Spiros Denaxas
**University College London**	Stephanie Wu
**University College London**	Tuankasfee Hama
**University College London**	Waty Lilaonitkul
**University College London**	Wojtek Banas
**University College London**	Yi Mu
**University College London**	Yohhei Hamada
**University College London**	Yoryos Lyratzopoulos
**University College London / DATAMIND**	David Osborn
**University Hospital of North Midlands**	Arun Pherwani
**University of Aberdeen**	Mary Joan Macleod
**University of Birmingham**	Sarah Wang
**University of Birmingham and Queen Elizabeth Hospital Birmingham**	Mark Thomas
**University of Bradford**	Alejandro Gonzalez-Aquines
**University of Bristol**	Arun Karthikeyan Suseeladevi
**University of Bristol**	Ben Gibbison
**University of Bristol**	Daniel Fudulu
**University of Bristol**	Dann Mitchell
**University of Bristol**	Deborah Lawler
**University of Bristol**	Eleanor Walsh
**University of Bristol**	Elsie Horne
**University of Bristol**	Elvis Annan
**University of Bristol**	Ewan Walker
**University of Bristol**	Falinyi Samson
**University of Bristol**	Gianni Angelini
**University of Bristol**	Jeremy Chan
**University of Bristol**	John Macleod
**University of Bristol**	Jonathan Sterne
**University of Bristol**	Joseph Okeke
**University of Bristol**	Katharine Looker
**University of Bristol**	Livia Pierotti
**University of Bristol**	Luisa Zuccolo

Institution	Member Name
**University of Bristol**	Marwa Al Arab
**University of Bristol**	Massimo Caputo
**University of Bristol**	Mercy Nthiwa
**University of Bristol**	Neil Davies
**University of Bristol**	Paul Madley-Dowd
**University of Bristol**	Rachel Denholm
**University of Bristol**	Rochelle Knight
**University of Bristol**	Rupert Payne
**University of Bristol**	Shubhra Sinha
**University of Bristol**	Tim Dong
**University of Bristol**	Tom Palmer
**University of Bristol**	Venexia Walker
**University of Bristol**	Yueying Li
**University of Bristol**	Yvonne Nartey
**University of Cambridge**	Alexia Sampri
**University of Cambridge**	Angela Wood
**University of Cambridge**	Carmen Petitjean
**University of Cambridge**	Chriselda Oliver
**University of Cambridge**	Elena Raffetti
**University of Cambridge**	Elias Allara
**University of Cambridge**	Emanuele Di Angelantonio
**University of Cambridge**	Eoin McKinney
**University of Cambridge**	Eric Harshfield
**University of Cambridge**	Fabian Falck
**University of Cambridge**	Genevieve Cezard
**University of Cambridge**	Hannah Harrison
**University of Cambridge**	Haoting Zhang
**University of Cambridge**	Holly Pavey
**University of Cambridge**	Isabel Walter
**University of Cambridge**	Jasmine Kiley
**University of Cambridge**	Jessica Barrett
**University of Cambridge**	John Danesh
**University of Cambridge**	Lisa Pennells

Institutions party to the Data Sharing Agreement with NHS England for access to the data in the Secure Data Environment service for England for CVD-COVID-UK/COVID-IMPACT are: Imperial College London, King's College London, London School of Hygiene & Tropical Medicine, Swansea University, University College London, University of Bristol, University of Cambridge, University of Dundee, University of Glasgow, University of Leicester, University of Liverpool, University of Manchester, University of Nottingham, University of Oxford, University of Sheffield, University of Southampton.

CVD-COVID-UK / COVID-IMPACT Consortium Members (03/04/2026)

British Heart Foundation Data Science Centre
Led by Health Data Research UK

Institution	Member Name
University of Cambridge	Lois Kim
University of Cambridge	Matilda Pitt
University of Cambridge	Mayank Dalakoti
University of Cambridge	Megan Ritson
University of Cambridge	Mike Inouye
University of Cambridge	Millie Zhou
University of Cambridge	Pearl Liu
University of Cambridge	Robert Fletcher
University of Cambridge	Rutendo Mapeta
University of Cambridge	Salma Chaudhury
University of Cambridge	Samantha Ip
University of Cambridge	Spencer Keene
University of Cambridge	Stelios Boulitsakis Logothetis
University of Cambridge	Stephen Kaptoge
University of Cambridge	Tom Pape
University of Cambridge	Wen Shi
University of Cambridge	Xilin Jiang
University of Cambridge	Xiyun Jiang
University of Cambridge	Yanfan Li
University of Dundee	Atanu Bhattacharjee
University of Dundee	Daniel Morales
University of Dundee	Huan Wang
University of Dundee	Ify Mordi
University of Dundee	Samira Bell
University of Edinburgh	Alan Carson
University of Edinburgh	Alice Hosking
University of Edinburgh	Athina Spiliopoulou
University of Edinburgh	Baljean Dhillon
University of Edinburgh	Carlos Sanchez Soriano
University of Edinburgh	Caroline Jackson
University of Edinburgh	Claire Tochel
University of Edinburgh	Dave Yeung
University of Edinburgh	Gavin Chapman

Institution	Member Name
University of Edinburgh	Gwenetta Curry
University of Edinburgh	Helen Colhoun
University of Edinburgh	Joe Mellor
University of Edinburgh	Kelly Fleetwood
University of Edinburgh	Laura Sherlock
University of Edinburgh	Luke Blackbourn
University of Edinburgh	Michelle Williams
University of Edinburgh	Miguel Bernabeu Llinares
University of Edinburgh	Rebecca Reynolds
University of Edinburgh	Richard Chin
University of Edinburgh	Steven Kerr
University of Edinburgh	Tim Wilkinson
University of Edinburgh	Verónica Cabreira
University of Edinburgh	William Berthon
University of Edinburgh	William Whiteley
University of Exeter	John Dennis
University of Exeter	Kerry Pearn
University of Exeter	Michael Allen
University of Glasgow	Angela Henderson
University of Glasgow	Clea du Toit
University of Glasgow	Colin Berry
University of Glasgow	Craig Melville
University of Glasgow	Dennis Tran
University of Glasgow	Filip Sosenko
University of Glasgow	Frederick Ho
University of Glasgow	Jill Pell
University of Glasgow	Jocelyn Friday
University of Glasgow	John Cleland
University of Glasgow	Naveed Sattar
University of Glasgow	Ninian Lang
University of Glasgow	Salil Deo
University of Glasgow	Sandosh Padmanabhan
University of Glasgow	Terry Quinn

Institution	Member Name
University of Leeds	Charlotte Sturley
University of Leeds	Jianhua Wu
University of Leeds	Jonathan Batty
University of Leeds	Marlous Hall
University of Leeds	Ramesh Nadarajah
University of Leeds	Tamara del Toro
University of Leicester	Anna Hansell
University of Leicester	Anvesha Singh
University of Leicester	Cameron Razieh
University of Leicester	Claire Lawson
University of Leicester	Clare Gillies
University of Leicester	Elpida Vounzoulaki
University of Leicester	Francesco Zaccardi
University of Leicester	Iain Squire
University of Leicester	Kamlesh Khunti
University of Leicester	Matthew Bown
University of Leicester	Muhammad Rashid
University of Leicester	Sharmin Shabnam
University of Leicester	Shirley Sze
University of Leicester	Tom Yates
University of Leicester	Yogini Chudasama
University of Lisbon	João Malato
University of Liverpool	Andrew Mason
University of Liverpool	Benedict Michael
University of Liverpool	Caroline Dale
University of Liverpool	David Hughes
University of Liverpool	Francesca Zaccagnino
University of Liverpool	Giulia Ballabio
University of Liverpool	Ike Chukwudi
University of Liverpool	Maria Sudell
University of Liverpool	Mark Green
University of Liverpool	Munir Pirmohamed
University of Liverpool	Orla Hilton

Institutions party to the Data Sharing Agreement with NHS England for access to the data in the Secure Data Environment service for England for CVD-COVID-UK/COVID-IMPACT are: Imperial College London, King's College London, London School of Hygiene & Tropical Medicine, Swansea University, University College London, University of Bristol, University of Cambridge, University of Dundee, University of Glasgow, University of Leicester, University of Liverpool, University of Manchester, University of Nottingham, University of Oxford, University of Sheffield, University of Southampton.

CVD-COVID-UK / COVID-IMPACT Consortium Members (03/04/2026)

British Heart Foundation Data Science Centre
Led by Health Data Research UK

Institution	Member Name
University of Liverpool	Pardis Biglarbeigi
University of Liverpool	Reecha Sofat
University of Liverpool	Rohan Takhar
University of Liverpool	Ruwanthi Kolamunnage-Dona
University of Liverpool	Stephen McKeever
University of Manchester	Bernard Keavney
University of Manchester	Craig Smith
University of Manchester	David Jenkins
University of Manchester	Eva Henning
University of Manchester	Evan Kontopantelis
University of Manchester	George Tilston
University of Manchester	Glen Martin
University of Manchester	Hector Chinoy
University of Manchester	Joseph Firth
University of Manchester	Lana Bojanić
University of Manchester	Matthew Sperrin
University of Manchester	Max Lyon
University of Manchester	Maya Buch
University of Manchester	Ruth Norris
University of Manchester	Sarah Steeg
University of Manchester	Simon Williams
University of Manchester	Steven Zhao
University of Manchester	Zenas Yiu
University of Milan	Federico Fama
University of Milan	Marta Colaneri
University of Newcastle	Camille Carroll
University of Newcastle	Charlotte Parbery-Clark
University of Newcastle	Dexter Canoy
University of Newcastle	Precious Onyeachu
University of Nottingham	Fiona Pearce
University of Nottingham	Laila Tata
University of Nottingham	Ralph Akyea
University of Nottingham	Stephanie Lax

Institution	Member Name
University of Oxford	Aashna Uppal
University of Oxford	Akshay Shah
University of Oxford	Antonella Delmestri
University of Oxford	Antony Palmer
University of Oxford	Ben Goldacre
University of Oxford	Ben Lacey
University of Oxford	Dani Prieto-Alhambra
University of Oxford	Eva Morris
University of Oxford	Gardash Bakhishli
University of Oxford	George Nicholson
University of Oxford	Hayley Evans
University of Oxford	James Sheppard
University of Oxford	Joseph Kamtchum Tatuene
University of Oxford	Kazem Rahimi
University of Oxford	Linxin Li
University of Oxford	Lucy Wright
University of Oxford	Marta Pineda Moncusi
University of Oxford	Mohammad Mamouei
University of Oxford	Nick Hall
University of Oxford	Parag Gajendragadkar
University of Oxford	Paula Dhiman
University of Oxford	Qingze Gu
University of Oxford	Ramla Khan
University of Oxford	Raph Goldacre
University of Oxford	Richard Williams
University of Oxford	Sara Khalid
University of Oxford	Seb Bacon
University of Oxford	Seyed Alireza Hasheminasab
University of Oxford	Shishir Rao
University of Oxford	Xiaomin Zhong
University of Oxford	Zeinab Bidel Taleshmekaeil
University of Oxford/KU Leuven	Nathalie Conrad
University of Plymouth	Marie-Louise Zeissler

Institution	Member Name
University of Sheffield	Norman Briffa
University of Sheffield	Suzanne Mason
University of Sheffield	Tim Chico
University of Southampton	Nazrul Islam
University of Strathclyde	Amanj Kurdi
University of Strathclyde	Kim Kavanagh
University of Strathclyde	Marion Bennie
University of Strathlcyde	Tanja Mueller
University of Warwick	Harry Wilde
University of Warwick	Majel McGranahan
University of York	Christina van der Feltz-Cornelis
University of York	Han-I Wang
University of York	Lorna Fraser
Wellcome Sanger Institute	Tapiwa Tungamirai

Institutions party to the Data Sharing Agreement with NHS England for access to the data in the Secure Data Environment service for England for CVD-COVID-UK/COVID-IMPACT are: Imperial College London, King's College London, London School of Hygiene & Tropical Medicine, Swansea University, University College London, University of Bristol, University of Cambridge, University of Dundee, University of Glasgow, University of Leicester, University of Liverpool, University of Manchester, University of Nottingham, University of Oxford, University of Sheffield, University of Southampton.
